# Methods for analysing patient flow between care settings: a systematic review

**DOI:** 10.1136/bmjopen-2025-115172

**Published:** 2026-08-28

**Authors:** Fan Yang, Marianne Sundlisæter Skinner, Sujan Rijal, Alan Lovell, Lisa Victoria Burrell, Maren Kristine Raknes Sogstad, Rob Anderson

**Affiliations:** 1 Department of Health Sciences in Gjøvik, Norwegian University of Science and Technology, Gjøvik, Norway; 2 Peninsula Technology Assessment Group, University of Exeter Medical School, University of Exeter, Exeter, UK; 3 Evidence Synthesis and Modelling for Health Improvement (ESMI), University of Exeter Medical School, University of Exeter, Exeter, UK

**Keywords:** STATISTICS & RESEARCH METHODS, Change management, HEALTH SERVICES ADMINISTRATION & MANAGEMENT, Organisation of health services, Health Services

## Abstract

**Abstract:**

**Objectives:**

To identify and appraise methods used to analyse patient flow across multiple care settings, and to describe how these methods have been applied to address health service research questions.

**Design:**

We searched MEDLINE, EMBASE, Web of Science databases and Google Scholar search engine between 1 January 2013 and 10 June 2026 using a systematic search strategy to identify the relevant literature for this review. Descriptive statistics and narrative synthesis were used to characterise the included papers.

**Results:**

Twenty-four studies met the inclusion criteria. Seven distinct methods and five health service research topics were identified. Most studies employed descriptive statistics and group comparison tests to provide a general description of the system. Discrete event simulation was more frequently used to simulate patient movement between settings. Furthermore, patient transfer volume and outcome, as well as capacity planning, were the most commonly studied health service research topics. This review highlights effective approaches—statistical analysis, mathematical modelling and computer simulation—for studying patient flow across care settings.

**Conclusions:**

Statistical analysis, mathematical modelling and computer simulation offer complementary approaches for analysing patient flow between care settings. Each method has distinct strengths and limitations. Applying more than one method may provide a richer understanding of patient flow. Future research may focus on comprehensive patient flow analysis, particularly in managing chronic conditions and non-communicable diseases in interconnected care settings and organisations.

**STRENGTHS AND LIMITATIONS OF THIS STUDY**

- Systematically overview and appraise methods for analysing patient flows between care settings or organisations.
- Restriction to English-language studies from 2013 onwards may have led to omission of relevant studies.
- The search was limited to health and general scientific databases and may have limited identification of some relevant methodological studies.

## Introduction

 Quality healthcare delivery within a treatment setting depends on patients attending or being transferred to the ‘right place’ at the ‘right time’.^1^ Such care is also contingent upon efficient patient flow, defined as having the optimal number of patients transferred through a healthcare facility without delays or resource waste.^1–4^ It is crucial to match available resources to clinical needs to improve patient flow. However, rising demand and persistent workforce shortfalls continue to challenge timely care delivery, forcing health systems to innovate and reallocate scarce resources to improve patient flow, across both single and multiple healthcare settings.^5^

The increasingly complex and interconnected healthcare landscape also drives the need to improve patient flow between care settings. Countries have been developing new care models such as intermediate-based, community-based and team-based care to bring services closer to the community and respond to the demand in a timely manner to make affordable care accessible. These new services and models inevitably introduce new challenges in coordinating patient flow among multiple care settings and levels.^6^ Various management approaches have been developed to address the complexity of coordinating patient flow across organisational boundaries, including care pathways that standardise patient journeys across different providers and care trajectories that track collaboration paths through multiple caregivers. Furthermore, the COVID-19 pandemic underscored the interconnected and interdependent nature of current health systems, particularly in efficiently managing patient flow between different care levels. This becomes particularly salient against the backdrop of constraints of limited capacity and resources, while systems face an increase in demand owing to disease transmission.^7^

Most studies on improving patient flow within emergency departments, primary healthcare centres and other community-based care units have reported reduced waiting times and increased efficiency for high volumes of patients.^8–10^ Examples of system-wide collaboration have introduced patient flow management systems to track patient flow across regions, enabling providers and decision-makers to maintain accurate oversight of patient flow, demand and capacity while improving patient outcomes and delivery efficiency.^5^ Given the potential benefits of improved patient flow, patient flow analysis is required to better understand current flow and care delivery, ultimately enabling practitioners and system managers to implement flow improvement actions to improve care.

Over the last two decades, there has been growing research interest in analysing and simulating patient flow to understand how current services are or may be delivered. One review identified six research techniques to analyse patient flow to highlight areas for improvement.^11^ These methods include analysing routinely collected data on service usage, capacity and workflow planning, computer modelling and simulation, failure mode and effects analysis, systematic feedback from staff and ethnographic observations.^11^ Most existing reviews have focused on applying a single modelling technique within a specific care setting, such as using machine learning in hospitals,^12^ computer simulations in emergency departments^13^ or artificial intelligence in mental health inpatient units^14^ to simulate patient flow. A systematic review of studies (published up to 2016) analysed operational research methods for patient flow modelling in community care settings. The authors noted that few models have considered patient flows between different community services, highlighting a significant gap in understanding patient flow through multiple services.^15^

This focus on single settings also highlights that much of the existing research on patient flow emphasises process-oriented outcomes, such as efficiency gains or improved throughput, without necessarily linking these improvements to broader system goals. Although improving patient flow can lead to more efficient use of resources within individual services, the downstream net effects from a system or societal perspective are harder to demonstrate. Some evidence suggests that even a relatively small increase in downstream community capacity can reduce pressure on acute bed bases, creating system-wide benefits.^16^ This highlights the importance of adopting a ‘system’ perspective and recognising interdependencies within the system. While the fundamental methods for patient flow analysis could be applied within a single setting, analyses spanning multisettings introduce additional challenges, such as integrating data across organisations,^17^ managing transitions of care and assessing system-level impacts, all of which are directly relevant to the strengths and limitations of these methods. Given the increasing complexity of healthcare systems, advancing our understanding of alternative methods for analysing patient flow between care settings may help policymakers and practitioners understand system interdependencies and support better service integration.

### Conceptualising patient flow between care settings

There is growing recognition that healthcare functions as complex adaptive systems (CAS), understood as “a collection of individual agents with freedom to act in ways that are not always totally predictable, and whose actions are interconnected so that one agent’s actions change the context for other agents."^18^ From this perspective, patient flow emerges from interactions among patients, healthcare professionals, organisations, technologies and policies, and changes in one part of the system can reverberate across others. This perspective is particularly relevant in the context of integrated care, which aims to reduce fragmentation in healthcare delivery through clinical, professional, organisational and system integration.^19^ Analysing patient flow between care settings thus sits firmly within a complexity perspective, in which system behaviour reflects adaptive responses to signals and constraints originating both within and beyond the health system.^20^

From a complex systems viewpoint, achieving normative and functional integration is not simply improving communication or adding capacity, but consequences of coordinating interdependent activities across distributed parts of the health system.^19^ Because interactions between agents are nonlinear and context-dependent in the CAS, overall system behaviour cannot be inferred by aggregating the effects of individual components, and outputs are not necessarily proportional to inputs.^18 21^ This viewpoint contrasts with much healthcare discourse, where care is conceptualised via a linear patient trajectory in the integrated care and transitions between care settings are assumed predictable.^22 23^ Conceptualising patient flow as a system-level phenomenon instead foregrounds the dynamic and adaptive work required to coordinate care across organisational boundaries in the contemporary integrated care system.

This review aims to synthesise evidence on the methods applied to patient flow analysis between care settings. Specifically, it aims to answer the question: What are the main methods used for patient flow analysis to address health service research questions? It also addresses the subquestion: What are the relative strengths and limitations of these methods in answering different research or evaluation questions? We followed the broad principles and guidance from the Centre for Reviews and Dissemination for undertaking reviews in healthcare^24^ and report the work in line with Synthesis Without Meta-analysis in systematic reviews: reporting guideline (SWiM) guidance where relevant.^25^

## Method

### Search strategy

In discussions with the two reviewers (FY and RA), an information specialist (AL) developed a search strategy to identify studies relevant to the two review questions. The search included three databases (PubMed/MEDLINE, Embase, Web of Science) and Google Scholar search engine. The review was restricted to studies published from 2013 to 2026 to maintain focus and ensure relevance to contemporary health service research questions. We did not include non-English studies due to the lack of translation capacity, and including non-English literature could have introduced inaccuracies in data extraction and synthesis. This review focuses on patient ‘movement’ between various care facilities; hence, ‘patient flow’ was the primary search term. For more comprehensive coverage, we incorporated numerous synonyms of flow during the search phase to account for potential variations in the terms used (eg, care flow, patient/care trajectory and patient/care pathways). The search was limited to studies and reviews with clearly stated patient flow between two or more settings and organisations. For the purpose of this review, we included studies that collected, analysed or applied real-world data to describe, evaluate or model patient flow between two or more settings or organisations. Studies that focused solely on theoretical frameworks, conceptual models or qualitative mapping of patient journeys were excluded. This distinction ensures that our review emphasises studies with a direct connection to observed or measured patient flows and grounded in real-world settings. For example, within system dynamics methods, causal loop diagrams were excluded unless they were integrated with quantitative modelling approaches, such as stock-and-flow models or parameterised simulations. Search strategy and protocol are available in Supplementary Information (see online supplemental information 1, 2).

### Study selection

**Figure 1:**
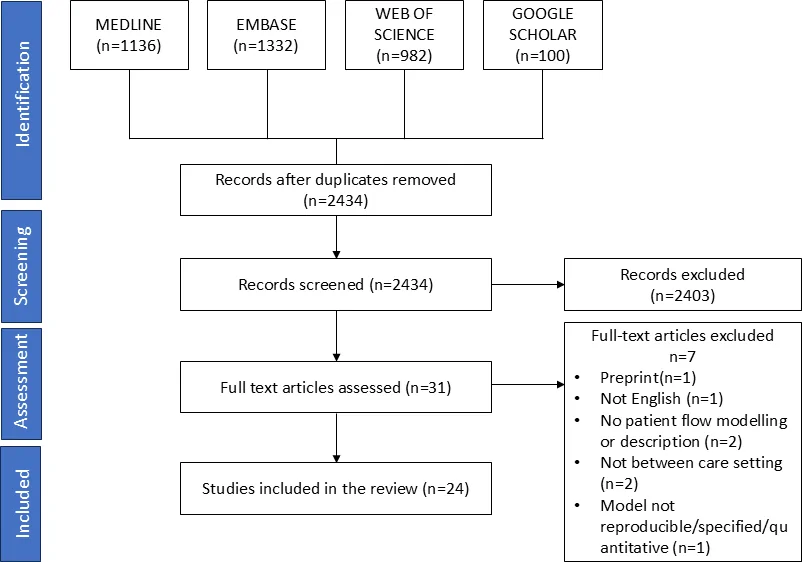
PRISMA study selection flow diagram. PRISMA, Preferred Reporting Items for Systematic Reviews and Meta-Analyses.

We searched for studies that referenced patient movements or transitions between different care settings, regardless of the terminology used. Figure 1 shows that 2434 records were identified from the database searches after duplicates were removed. The records were imported to the web-based systematic review programme (Rayyan, https://www.rayyan.ai/)^26^ to be shared among authors for screening. During the identification stage, three authors (FY, RA and SR) screened the first 100 titles and abstracts based on the initial inclusion criteria to clarify and reach a consensus on the application of the inclusion and exclusion criteria (table 1). FY and SR independently screened all titles and abstracts and discussed uncertain decisions with RA. FY and RA independently screened the full texts of 31 articles using a screening form developed by FY, checked the extracted information and decided on the final inclusion records. 24 studies were included after excluding seven full-text articles at the full text stage.

**Table 1:** Selection inclusion and exclusion criteria

	Inclusion criteria	Exclusion criteria
1	Studies published from 2013 onwards	
2	In English language	
3	Studies that include patient flow analysis between any two or more care settings/organisations, such as hospitals, primary care clinics, outpatient/specialist clinics, long-term care facilities or home healthcare. AND Studies that analyse patient flow between care settings using any method, such as workflow analysis, process mapping, time and motion studies, simulation modelling, data analysis, etc.	Studies that only analyse patient flow within one care setting, rather than between different care settings. OR Studies that do not involve patient flow analysis.
3a	OR are: Reviews or comparative studies on different methods for analysing patient flow (including reviews that analyse patient flows between care settings and organisations)	Protocols

No existing validated quality appraisal tool was suitable for methodological studies of patient flow analysis, so we developed a bespoke domain-based checklist. This checklist assesses: (1) conceptual clarity (whether patient flow and movement between care settings are clearly defined), (2) method specification (whether the analytical method is described in sufficient detail, with underlying assumptions and model structure clearly reported), (3) data and setting (clarity about data sources, units and time horizon) and (4) reporting and interpretability of results. Each domain was scored on a 3-point scale: 0=not reported, 1=partially reported, 2=clearly reported, giving a maximum total score of 8. Studies scoring 6 or above were considered to have adequate methodological reporting. Quality scores were used to contextualise rather than exclude studies from the synthesis. Quality assessment was conducted by one reviewer (FY) and independently verified by a second reviewer (RA); discrepancies were resolved through discussion.

### Data extraction, analysis and reporting

**Figure 2:**
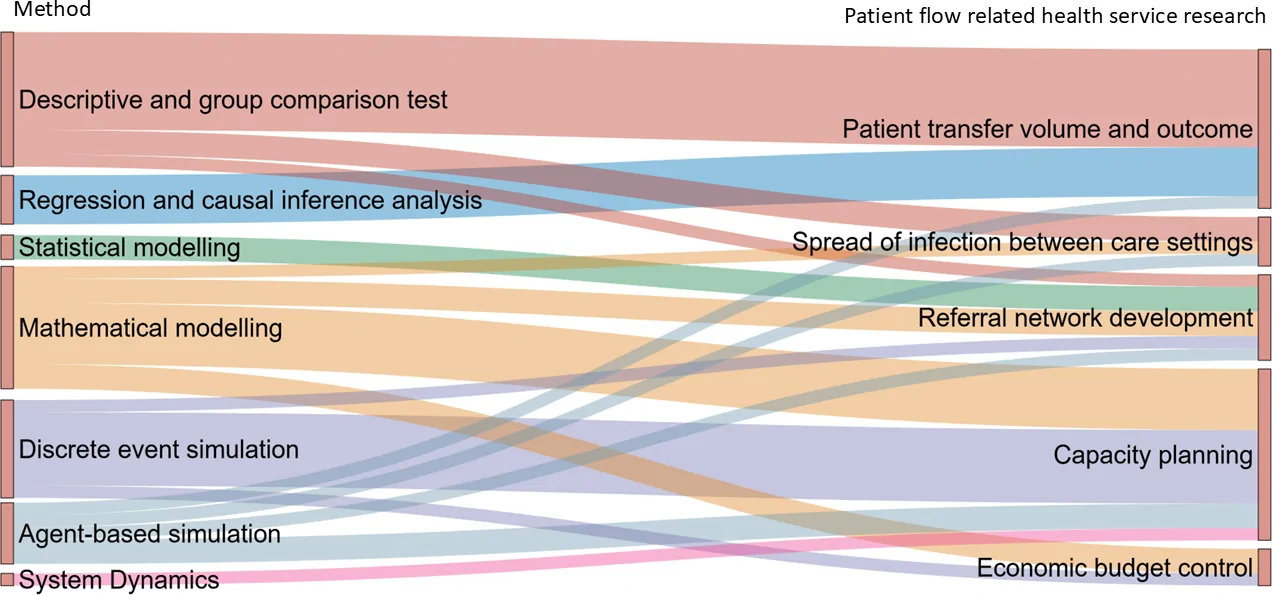
Sankey plot of the analysis methods and patient flow-related health service research topic.

Key information on each included study was extracted and recorded by one reviewer and independently checked by a second reviewer. Discrepancies were resolved through discussion and consensus. The information includes publication year, authors, objective/aims, patient flow-related health service topics, involved care settings, methods of analysing patient flow and method output (online supplemental information 3). A Sankey plot was created to visualise the relationship between the identified analysis method and the main patient flow-related health service topic of each study (figure 2). The Sankey plot highlights the various methods used to address a common health-service-related problem and identifies whether a specific method has been applied to any particular service-related issue.

The results are summarised and synthesised based on the design and findings of each study.

### Patient and public involvement

None.

## Results

**Figure 3:**
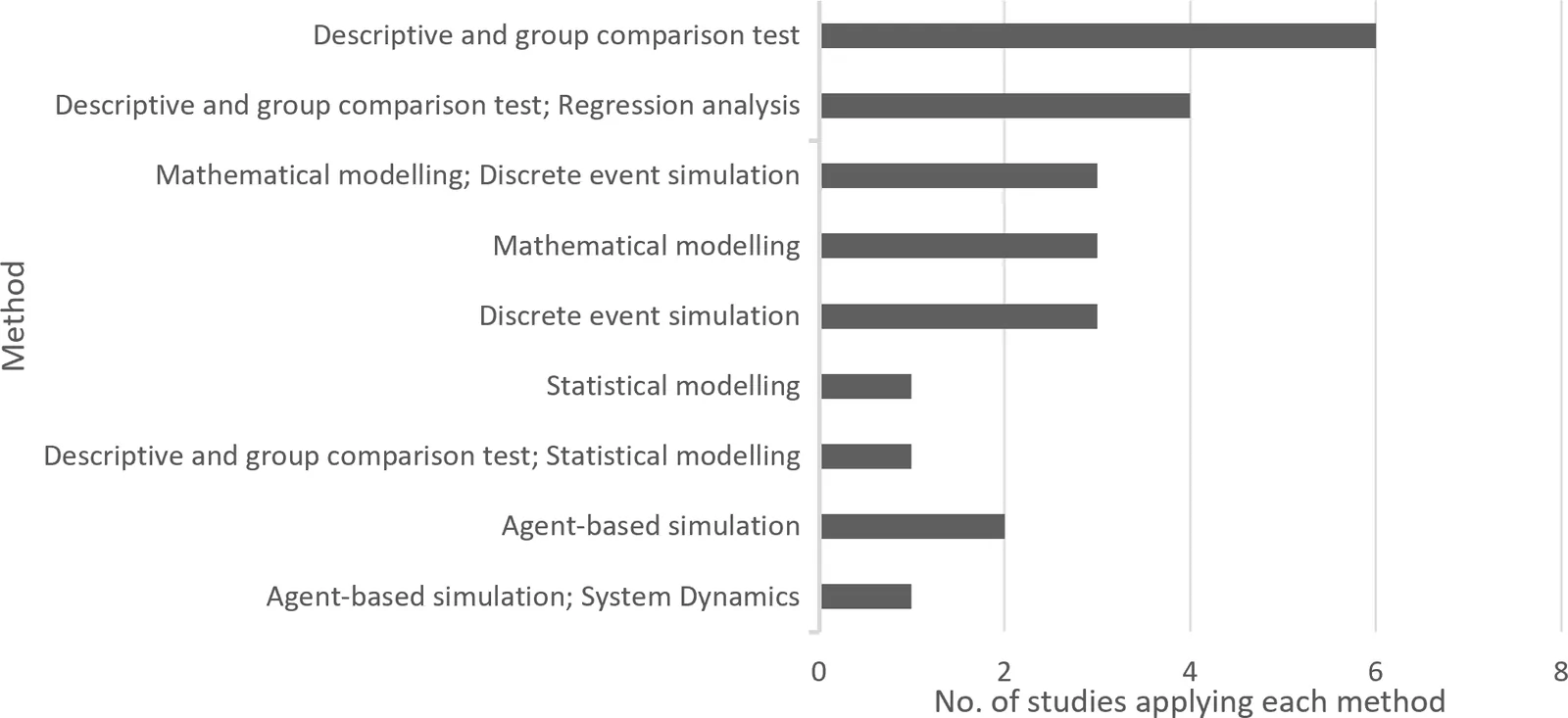
Frequency of method combinations applied in related studies.

Seven discrete methods and nine combinations of these methods were identified from the included studies (figure 3). The most frequently used were descriptive and group comparison tests (11 studies), mathematical modelling (6 studies) and discrete event simulation (6 studies). Notably, descriptive and group comparison tests were commonly combined with other methods, such as regression analysis (in four studies) or statistical modelling. Three articles combined mathematical modelling with discrete event simulations. Agent-based simulation was used alone in two studies and in combination with system dynamics modelling.

**Figure 4:**
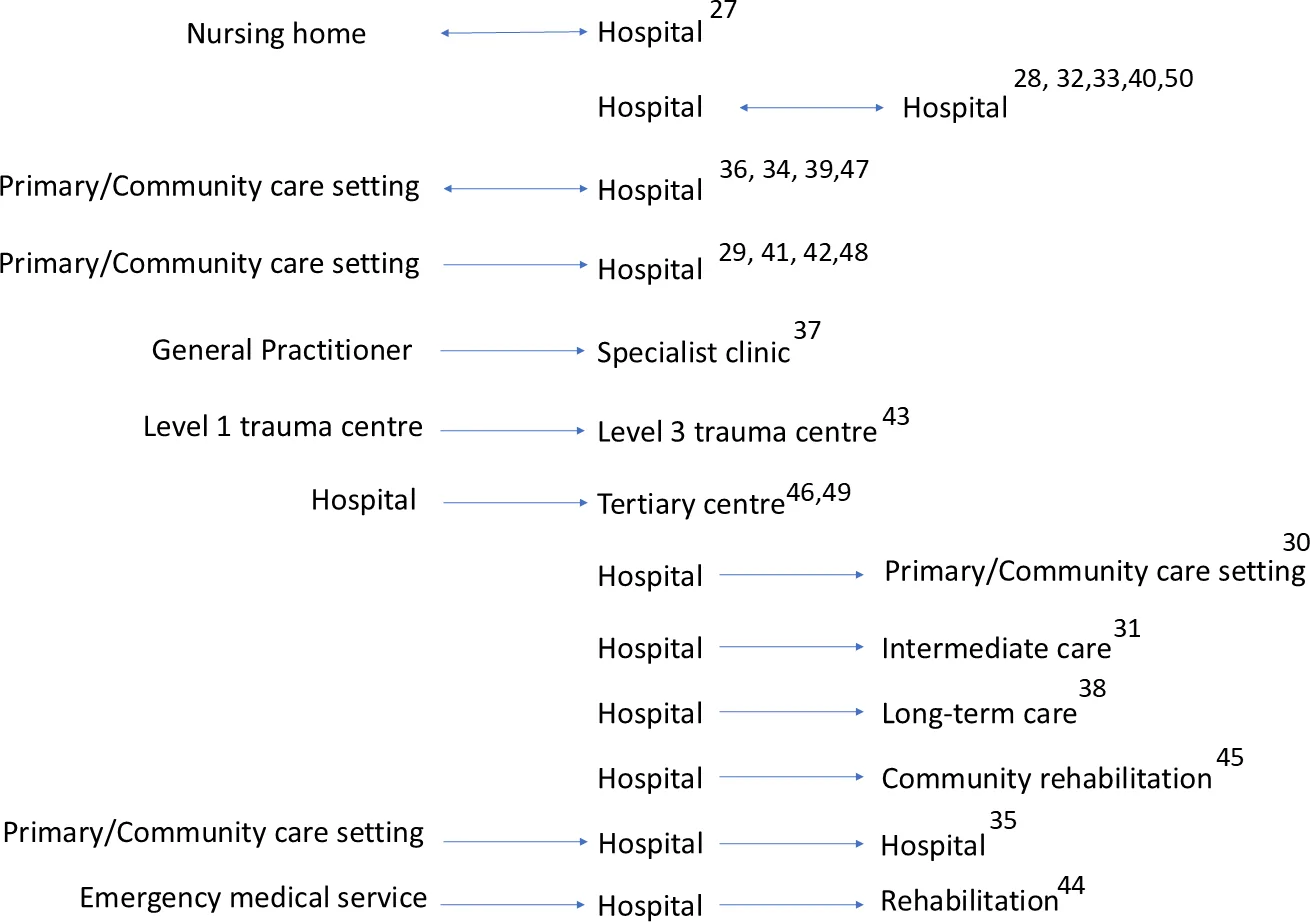
Patient flow between different care settings in 24 included studies.

Figure 4 maps the care settings involved and the directions of patient movement across the included studies. Most studies analysed flows to, from, or between different hospitals (or specialist services), highlighting hospitals as the central hubs and the primary focus of research. The majority of studies (14 studies) analysed flows in a single direction (eg, from hospitals to long-term care facilities). They mapped pathways illustrate both transitions to higher intensity care (eg, from primary care to hospital care) and transitions toward community rehabilitation or primary care.

### Methods used to analyse patient flow

Figure 2 maps the methods identified across the included studies and the frequency with which each was applied to different health service-related problems. For the synthesis, descriptive and group comparison tests together with regression analysis are grouped as ‘statistical analysis’; statistical modelling and mathematical modelling are grouped as ‘mathematical modelling’; and discrete event simulation (DES), agent-based simulation (ABS) and system dynamics modelling (SDM) are grouped as ‘computer simulation’.

#### Statistical analysis

In studies using statistical analysis, patient flow was quantified as the total number of patients transferred or moved, as well as the related ratios or percentages between care settings. Descriptive and group comparison tests provided summary insights into distinct patient groups. Regression analysis can then identify factors that are potentially associated with the number and volume of patients transferred and quantify the magnitude of the relationship.

Except for studies that solely aimed to compare or describe patient movement numbers or volumes,^27–31^ descriptive statistics and group comparison tests were frequently used along with other methods during the initial stage of analysis to summarise the characteristics of the context. Four studies in the review conducted regression analysis,^32–35^ and one used statistical modelling after descriptive statistical analysis.^36^ The three health service-related topics were fully or partially analysed using descriptive statistics and group comparison tests, including patient transfer volume and outcomes,^28 29 31–33 35 37 38^ analysing the spread of infections between care settings^27 30^ and informing the development of a referral network.^36^ Regression analysis and causal inference approaches focused on patient flow and interhospital transfer decisions, aiming to help identify the right patients to transfer.^33–35^ Using the difference-in-differences approach, Yuan *et al* assessed the impact of the policy on patient flow by analysing the admission ratios of primary care institutions and hospitals and evaluating the effectiveness of their policy to integrate primary and secondary care.^32^

#### Mathematical modelling

Mathematical modelling is the process of representing and describing real-world events using mathematical symbols and/or equations to understand a problem and make predictions.^39^ Six studies used mathematical models to identify and simulate patient flow between care levels and organisations.^40–45^ These studies analysed patient flow to determine the imbalance between system capacity and patient demand in the healthcare system with the aim of determining the optimal capacity. Statistical modelling is a branch of mathematical modelling that uses statistical techniques and typically incorporates uncertainties.^36 46^

Four patient-flow-related topics were fully or partially analysed using mathematical modelling (figure 2). Five studies discussed capacity planning, with two specifically examining capacity planning under economic budget control. Wood *et al* developed a model by applying the queueing theory to estimate the cost-optimal levels of delayed transfers.^40^ Onen-Dumlu *et al* also used the queuing theory to model care demand and cost-optimal capacity between acute and intermediate care settings.^41^ Song *et al* developed a model to simulate patient flow distribution within a hierarchical healthcare system, calculating throughput, patient length of stay, the utilisation rate and the ‘leave without being seen’ rate.^42^ Piotrowska *et al* developed a mathematical model to simulate the spread of Multidrug-resistant Enterobacteriaceae (MDR-E) within a regional health network.^43^ Finally, Qiu *et al* proposed a simulation-based optimisation method to evaluate the optimal patient flow distribution between communities and general hospitals.^44^

Two applications of statistical modelling have evaluated interhospital patient flow by modelling patient transfer networks. Caimo *et al* introduced Bayesian exponential random graph modelling to assess the regional referral propensity between hospitals and enhance parameter estimation accuracy through a fully probabilistic analytical framework.^36^ Justice *et al* sought to design a better imputation algorithm to address missing values in interhospital transfer data. Both studies applied a probabilistic analytical framework, which improved the accuracy of their analyses.^46^

#### Computer simulation

Computer simulations can estimate the dynamic responses of systems under various scenarios and assumptions by replicating real-world systems that cannot be easily applied or evaluated in practice.^47^ Three computer simulation methods were identified: DES, ABS and SDM. Patient flow was simulated by replicating system interactions and care delivery processes through predetermined hypothetical parameters, such as indicators of capacity and demand. Some studies may conduct mathematical modelling to develop analytical equations and formulas that define relevant variables, states and transitions for further testing in the computer simulation. Others proceed directly to simulation using empirical data for scenario testing. For example, Song *et al* provided a strong mathematical modelling foundation as the basis for subsequent DES.^42^

##### Discrete event simulation

DES is a method of modelling and simulating the behaviour and performance of a process, facility or system as a sequence of events occurring at particular points in time.^48^ It tracks patients as they move through various states (eg, between different care levels and organisations), with transitions between these states determined by discrete events (eg, hospital admissions). Six studies were found, all of which visualised the processes and interactions between interconnected systems. The transitions ranged from movements between community health centres and general hospitals,^42 44^ acute care to intermediate care settings,^41^ acute care hospitals to long-term care facilities^49^ and acute care wards to community rehabilitation.^50^

A queuing network is commonly applied in DES analysis of patient flow, therefore, the output measures are similar across different applications. For example, these simulation studies have estimated the capacity required to reduce discharge delays,^41 50^ improve throughput,^42 44^ optimise resource utilisation,^42 44 49^ control the length of stay^42^ and reduce patient waiting times.^49^

##### Agent-based simulation

ABS is a method of modelling a system as a collection of decision-making entities (the ‘agents’) interacting with one another, and that make decisions in accordance with a predetermined set of rules.^51^ This approach can exhibit complex behavioural patterns and imitate actual systems.^51^ Lee *et al* used ABS to track the transmission of drug-resistant bacteria and patients’ infected states through patient flow interactions between care settings and constructed a regional referral system by employing a transfer probability matrix between various healthcare facilities. The model also simulates how changes in capacity at one facility affect other regional healthcare facilities.^52^ Brice *et al* used the ABS to map the number of depressed patients with different severities and treatment statuses moving through care systems to calculate the capacity required. The model captured both the severity of patients’ depression, ranging from mild to severe and whether they were in treatment, post-treatment or not treated.^53^ The results of these ABS studies are related to tracking and measuring the prevalence of disease in the population.

##### System dynamics modelling

SDM is a simulation method used to understand how all objects in a system interact with one another. It enhances the comprehension of the structure and dynamics of complex systems and thus enables better decision-making.^54^ In this review, SDM was used in combination with ABS by Brice *et al* to capture both individual agent behaviour and system-level feedback dynamics simultaneously,^53^ a combination suited to problems where micro-level interactions produce macro-level patterns, such as infection transmission across a regional care network. The hybrid design allowed disease progression (via ABS) and operational patient flow (via SDM) to be modelled simultaneously.

### Patient flow-related health service research

The five health service research topics identified across the included studies—capacity planning (10 studies), patient transfer volumes and related outcomes (9 studies), informing the development of a patient referral network (5 studies), estimating the spread of infections between care settings (4 studies) and economic budget control (2 studies). These are visualised in relation to method use in figure 2. Although particular research topics can be studied using several methods, each approach has its own main problem-solving focus and set of characteristics. The methods are chosen precisely because their inherent strengths and weaknesses align with what the research question needs. It is common for one or two research topics to be addressed simultaneously in a single study.

Patient transfer volumes and outcomes were addressed exclusively using statistical analyses (figure 2). Descriptive and group comparison tests described or compared patient groups to measure patient transfer volumes between care settings. Regression and causal inference analyses aimed to compare patient outcomes under different patient flows,^31 33 34^ or identify factors associated with patient flow.^35^

Studies have focused on capacity planning, primarily aimed at stimulating the capacity and demand of the prevailing system. This helped to determine the optimal capacity under a hypothetical patient flow by evaluating performance measures, such as delays and wait times. Ten studies analysed capacity planning, four of which focused only on capacity planning using discrete event simulations^42 49 50 55^ combined with mathematical modelling.^42 50^ The remaining five studies cover two to three topics, including two on economic budget control.^40 41^ These two studies suggested that following capacity simulation, DES and mathematical modelling could be applied to further simulate costs and determine the most cost-effective capacity settings. Three studies analysed capacity planning using agent-based simulations, where capacity planning focused on strategic resource allocation in the face of increasing patient numbers and transmission.^52 53^ These two studies used agent-based simulations, which are well suited for scenarios involving interactions between healthcare settings, patients and diseases. Additionally, Lee *et al* discussed referral network development to better understand the spread of the disease during patient referral and transfer in the region.^52^ Qiu *et al* studied the two topics of referral network development and capacity planning using discrete events to evaluate patient flow distribution, measuring outcomes such as throughput, service utilisation and system profit.^44^

### Method comparison

Table 2 presents the method comparison concluded from the included literature.

**Table 2:** Features of methods used in analyses of patient flow

	Statistical analysis	Mathematical modelling	Computer simulation
Aim of the research	To estimate the infectious rate during disease transmission.^27 30^ To evaluate the accuracy of referral and unnecessary referrals.^28^ To reveal interhospital collaboration through patient transfer.^29^ To evaluate integrated policy reform on patient transfer.^32^ To compare patient outcome under different patient flow.^31 33 34^ To identify factors related to unnecessary transfers in a regional transfer network.^35^	To reveal interhospital collaboration networks through patient referral relations.^36 46^ To illustrate the false economy of eliminating DTOC.^40^ To estimate cost-optimal capacity.^40^ To reduce the spread of disease transmission under a regional network level.^43^	To estimate cost-optimal capacity.^41^ To estimate capacity for related demand of care.^41 49 50 53^ To simulate the spread and control of disease transmission.^52 59^ To simulate optimal patient flow distribution^42 44^ To capture depression progression.^53 60^
Output	Disease prevalence.^27^ Incidence of unnecessary transfers.^28 35^ Patient transfer numbers and percentages.^29 30 32^ Mortality rate.^31 33 34^ Length of stay.^34^	Referral propensity.^36^ Cost-optimal delayed patient transfer amount.^40^ Patient transfer network.^43 46^ Disease prevalence.^43^	Demand for care.^41 49 53^ Cost-optimal delayed patient transfer amount.^41^ Infection prevalence.^52 53^ Patient transfer network.^44 52^ Length of stay.^42 52^ Probability of readmission.^52^ Bed/service capacity.^49 50 52 53^ Leave without being seen rate.^42^ Throughput.^42 44^ Wait time.^49^ Resource utilisation.^49^ System profit.^44^ Probability of delay.^50^
Feature and possible application	Useful for generating basic understanding for current patient demographic and clinical characteristics.^27–35^ Using quasi-experiments can find the causal inference between policy or intervention and patient flow.^32^ Taking time-effect into consideration.^32^ Capture the disease prevalence and transmission.^30^ Reveal the relationship between patient flow and associated factors.^33–35^	Can incorporate assumptions for interhospital dependencies.^36 43^ The model can be generalised to other care settings.^36^ Basic theory (eg, queuing theory) can support further analysis.^40 43^ Useful tool to provide relationship between delayed discharge and marginal cost and capacity.^40^ Using network to estimate patient flow between facilities and related communities.^43^	Dynamically model the flow of individual patients across two care settings.^41^ The model can be generalised to other care settings.^41 42 52^ Useful tool to predict future care demand and resource allocation.^41 49^ Visualise the individual patient flow across two care settings.^41 42 44 49 50 52 53^ Allow stochastic variable in the model.^50 52^ Visualise the decision-making process through individual patient flow.^52^ Useful data-driven decision tool for decision-makers to identify where healthcare resources are needed.^49 50^ Integrate with other optimisation algorithm to improve model efficiency.^44^ Hybrid models can synchronise disease progression and patient flow in the system.^53^ Apply routinely to collect easily obtained administrative data.^50^
Limitations	The design and findings are strongly restricted to current data.^27 30 32 34^ The findings may not be generalised to other settings.^28 29 31 32^ Missing data results in bias.^31–33^	Have a computational burden in the parameter estimation and model selection.^36^ Lack of inferential approaches for imputing missing data.^36^ Have data limitations in parameter estimation.^40^ Did not classify patient demographics, consider all patients are standard patient, and this may cause bias in results.^43^	The model did not encompass the full complexity of the actual system, but simplifies it with assumptions.^41 52^ The outcome is restricted to the level of simplification of the real world and assumptions.^41 52^ Did not classify patient demographics, consider all patients are standard patient.^42 44^ Have data limitations in parameter estimation.^41 42 44 52 53^ Need adjustment to generalise to other contexts.^53^ Cannot predict performance metrics such as length of delay due to the lack of data.^50^
Visualisation	Seldom use visualisation for patient flow.	Seldom use visualisation for patient flow.	Apply flow chart for DES and ABS; stock-flow chart for SDM.

ABS, agent-based simulation; DES, discrete event simulation; DTOC, delayed transfers of care; SDM, system dynamics modelling.

Statistical analysis of patient flow offers several advantages. It is conducive to generate a foundational understanding of current patient demographics and clinical characteristics. Additionally, it can reveal the relationship between patient flow and its associated factors. The limitations of this method are that the design and findings are restricted to current data,^27 30 32 34^ missing data may result in bias^31–33^ and the findings may not be generalisable to other settings.^28 29 31^ Moreover, the studies that used statistical analysis seldom included patient flow visualisation in their study, which may inhibit readers’ understanding of how patients move across the system.

Models are a ‘simplified representation or description of a system or situation, used to improve understanding or decision making’.^56^ Mathematical modelling was established either on queueing theory, an epidemiological model, or a budget constraint model in the reviewed articles. Existing theories sometimes enable authors to make assumptions about the relationship between capacity and patient flow. Although studies have shown that mathematical modelling is usually conducted using computer simulations, this method provides a range of benefits. The strength of mathematical modelling lies in its capacity to formulate abstract questions related to patient flow using equations. The relationships between the variables can also be plotted. The limitation is that the data required for model calibration (ie, validation) ideally need to be from different sources, which is not always possible and this could affect the results and conclusions.^40 41^ Although mathematical models help simplify the relationships between variables, modelling itself involves a computational burden in parameter estimation and model selection.^36^

Computer simulation methods have limitation in that the sourcing of valid and reliable input parameters is limited, perhaps partly because of a lack of data affects the certainty of model outcomes.^41 53^ Another common problem when using computer simulation methods is that the simulation results depend closely on certain core assumptions regarding patient flow, capacity and referral.^41^ The effectiveness of computer simulations is often related to these core assumptions or hypotheses, which requires the model to contain sufficient detail to appropriately capture relevant issues without going into excessive detail that could complicate the system. Lee and colleagues mentioned in their discussion that they ignored some details to simplify the model, which could have affected their results.^52^ Song *et al* argued that because they did not include variations in individual patient characteristics (such as age, sex and other demographics), it may have obstructed the reflection of individual decision-making behaviours in patient flow allocation during capacity planning.^42^ These also reflect concerns in computer simulation regarding model validation and computation limitations in handling uncertainty. In particular, external validation requires real-world data, which is often under-described in practice. When simulations are conducted prior to system implementation, such data may not yet be available for validation.^41^ As a result, most studies rely on face validation, typically through expert review or workshops with healthcare professionals.^50 53^ Cross validation, meanwhile, is often difficult to achieve, as simulations are constructed under specific assumptions and may not generalise well when those assumptions or implementation settings change.^50 52^ It is also important to note that the computational burden can hinder the ability of simulation models to incorporate complexity and adequately account for uncertainty, which constrains their usefulness for informing decisions that require precise estimates of expected benefits.

The main strength of computer simulation methods for analysing patient flow is also notable that they model the entire system with its core elements, which provides a holistic view of how patients move through different parts of the service system over time. This applies especially to the connections between different care levels and organisations. Computer simulation methods provide decision-makers and managers with insights into which part(s) of the service provision require improvement and how patient flow will respond as capacity or referral/demand volume changes. Computer simulation methods can provide illustrative forecasts of how interventions might influence the flow prior to implementation in practice. Additionally, flow charts that visualise the entire computer simulation were effective in illustrating how the various components of the system worked together.

### Method selection and applicability

Method selection in practice is shaped not only by the research question but by the system conditions in which the analysis is conducted, including research aim, data requirement, output type, interpretability and modelling expertise required. Table 3 summarises these dimensions across the three method groups.

**Table 3:** Method selection guide for patient flow analysis between care settings

Dimension	Statistical analysis	Mathematical modelling	Computer simulation
System conditions	Data-rich, retrospective setting; questions focused on observed patterns rather than prospective scenarios.	Formal system structure; theoretical optimisation or analytical benchmarking needed.	Prospective planning; stochastic demand; complex multisetting systems.
Best-fit research aim	Describe or explain observed flows, associations or causal inference questions.	Formalise theoretical relationships; provide analytical foundation for simulation.	Test prospective scenarios; apply to complex system with agent interactions.
Data requirement	Routinely collected administrative data.	Aggregate parameters; theory-derived equations.	Patient-level or aggregate data; validated inputs.
Output type	Descriptive/explanatory	Theoretical benchmark	Scenario-based forecast
Interpretability to decision-maker	High – summary statistics and regression outputs are accessible.	Moderate – equations and model outputs require modelling literacy and interpretation support.	Moderate – output can be visually compelling but underlying assumptions require expert explanation.
Computational requirements	Low – moderate	Moderate – high	High
Feasibility in real-world settings	High – widely used in routine health service reporting.	Moderate – depends on availability of system parameters.	Low-moderate – resource-intensive; rarely implemented without dedicated operation researcher/analyst support.

Statistical analysis is most applicable when routinely collected administrative data are available and the primary aim is to describe current patient flows. Requiring minimal modelling assumptions and producing outputs easily interpretable to decision-makers, this descriptive analysis delivers foundational, firsthand insights into the current landscape. When moving from descriptive to regression analysis, the aim shifts beyond simple description to isolate the drivers or associated factors varying in patient flow. It yields empirically grounded insights that show decision-makers the underlying variables shaping the current landscape. Statistical analysis, while producing interpretable findings, was applied retrospectively in most cases and therefore informed understanding of existing systems rather than guiding prospective change.

Mathematical modelling is most applicable when a theoretical relationship between system variables needs to be formalised analytically, such as quantifying the relationship between capacity and total cost applying queueing theory^40^ or the dynamics of infection spread across a network.^43^ It is most powerful as a precursor to simulation, providing the structural equations and parameter estimates that anchor more complex models. Its outputs are theoretically precise but contingent on specified assumptions, and require a degree of relevant mathematical, epidemiological or economic modelling literacy for both construction and interpretation.

Computer simulation is most applicable when decision-makers need to test prospective ‘what-if’ scenarios before committing to service changes,^42^ and where stochastic variation in demand and capacity is central to the question.^41^ It demands investment in sourcing patient-level or aggregated data for model input parameter and validation. ABS and SDM extend this further to questions involving complex agent interactions or dynamic feedback. Across all simulation types, construction and interpretation also require certain expertise in mathematical, epidemiological or economic modelling literacy, as well as familiarity with specialist simulation software. In addition, DES studies show the strongest evidence of operational embedding in the included literature. Two studies describe models developed in collaboration with health service teams, with outputs explicitly intended to direct service evaluation and inform service planning decision.^41 50^

## Discussion

### Summary of findings

Our review identified seven methods which can be grouped into three broad categories: statistical analysis, mathematical modelling and computer simulation. These methods have been used to analyse patient flow between care settings and address a range of health service research problems. The dominance of statistical analysis reflects the relative accessibility of routinely collected administrative data across the included settings, while the prevalence of DES reflects the scenario-based nature of capacity planning questions that dominate the literature.

In general, each method involves inherent trade-offs that determine its suitability for different research objectives. Statistical analyses (descriptive and group comparison tests and regression analysis) trades explanatory power for simplicity and reliability, making them ideal for describing patient or system characteristics and providing comparisons between groups and outcomes. Mathematical modelling trades realism and flexibility for the ability to derive theoretical insights and generate analytical solutions. Modelling generates equations for the expected output, such as waiting time and length of stay under specified assumptions. Computer simulation methods trade computational efficiency and simplicity for complexity handling, allowing researchers to use predetermined parameters to visualise trends in the output under multiple scenarios.

The circular relationship between methods and research questions makes it challenging to generate relative strength comparisons, since methods are selected for their fit with specific research objectives. Instead, our review highlights how diverse methods complement each other: statistical analysis aims at describing and comparing, mathematical modelling aims at abstracting relations between key variables and computer simulation methods aims at prospective scenario testing and predicting system responses under different conditions.

### Heterogeneity and context-dependence

The wide variation in clinical context, healthcare system structure and modelling approach across the included studies is not merely background heterogeneity but also shapes method selection and the transferability of findings.

Clinical context shapes both the method selected and the scope of valid inference. Studies addressing retrospective questions using statistical analysis, such as transfer volume, infection prevalence and referral patterns, whose findings are valid for the described population but do not generalise beyond the study settings. Studies addressing prospective questions such as capacity planning and cost optimisation require simulation or mathematical modelling, whose validity depends on how accurately the disease pathway is represented in the model; for example, the stroke rehabilitation pathway^50^ and the depression care pathway.^53^ Transferability depends more on research question than method: findings about infection spread dynamics are more generalisable across different infectious diseases, because the underlying epidemiological relationships are less system-specific.

Healthcare system structure is an important contextual factor for transferability. Models are parameterised within a specific system type such as China’s tiered referral structure^42 44^ or UK National Health Service (NHS) integrated pathways,^40 41 53^ and carry embedded structural assumptions that may not hold elsewhere. Findings are most transferable to studies sharing a similar broad healthcare system context. Beyond this macro level, transferability also operates at the level of the specific care configuration studied. Models built around trauma and acute care pathway,^34 35^ intermediate care step-down services,^41^ long-term care networks,^49^ rehabilitation services^31 50^ or hierarchical referral tiers^42 44^ carry pathway-specific assumptions about patient acuity, transfer triggers and capacity norms that may not transfer to another setting within the same healthcare system.

Modelling approach also shapes the validity and transferability of findings independently of system context. Studies using hybrid approaches, combining mathematical modelling and DES, produce outputs whose validity depends on coherence of assumptions across both components, making them harder to validate than single-method studies. Studies using ABS or SDM produce outputs whose validity depends on whether agent rules and feedback structures were designed to reflect observed rather than assumed patient behaviour. These method-specific validity requirements mean that findings from one modelling approach cannot be straightforwardly compared with or applied alongside findings from a different approach.

### Limitations of the methods

Beyond the contextual variation that needs to be considered in method selection and comparison, methodological limitations exist regardless of system context and reflect variations in how patient flow studies are conducted and reported across all three method groups. First, external validity is consistently restricted across all method types. Statistical analysis findings describe the specific population and time period studied where the transfer rates, infection prevalence and referral patterns are system-specific and cannot be assumed to hold in a different setting or time period; mathematical models hold a better external validity for the modelling part because the modelling ideas are built on specific theory and pathway, such as queuing theory and infection pathway, but the concrete models are built on theoretical parameters derived from a specific system, for example, Wood *et al*’s cost-optimality threshold is specific to NHS cost structures and needs to be tweaked when applied to systems with different financing.^40^ Simulation models are parameterised for a certain system configuration and cannot be reliably applied to other systems with different capacity ratios, referral patterns or patient demographics without re-parameterisation. Across all three approaches, most included studies do not explicitly acknowledge these validity boundaries, which risks practitioners applying findings beyond their appropriate scope.

Second, reproducibility is a systematic concern across the included literature. Few included studies provide analytical code; full parameter sets or input data publicly available. For statistical analysis, replication requires access to the same restricted administrative datasets; for modelling and simulation studies, the level of methodological detail reported varied considerably, with several studies providing insufficient information to reconstruct the model independently. Consequently, these studies fall short of health research reproducibility standards, making independent verification difficult. However, the model structure can be partially inferred from reported equations and flow diagrams, providing a useful reference for researchers seeking to adapt or replicate these approaches in new settings.

Third, parameter uncertainty was addressed more thoroughly in simulation studies than in statistical analysis. Simulation studies more commonly provided scenario testing, varied arrival rates, length of stay and cost-capacity ratios to explore how outputs change under different conditions. In contrast, statistical studies rarely tested analytical choices such as covariate selection or handling of missing data against alternatives. That said, even within simulation studies, formal sensitivity analysis remained limited. Given that capacity planning decisions depend on understanding the range of plausible outcomes rather than a single projected value, more consistent reporting of parameter uncertainty across all method types would strengthen the evidence base.

Fourth, the gap between analytical outputs and policy implementation should be treated carefully. Statistical studies produce retrospective descriptions that are not directly actionable for prospective planning without additional modelling steps. Mathematical modelling produces theoretically grounded recommendations that are actionable at the strategic level, for example, Wood *et al* not only demonstrated the misleading nature of a ‘zero DTOC’ target but explicitly suggested managers to lessen required capacity through reducing inherent variability in the system.^40^ Even though the insights are valuable, the implementation needs to combine the reality conditions, as the authors pointed out, the model only covers Delayed transfer of care (DTOCs) attributable to NHS-funded community care. This suggests that the outputs may point a direction to the implementation, but the manager needs to take into account local condition. Simulation studies are more actionable, but most are still presented in the academic contributions without describing whether outputs informed real decisions. Only two studies mentioned the collaboration work with clinicians and managers in model development,^41 53^ which suggests a viable pathway for model validation and translating research findings into practice.

Fifth, data sources constrain both validity and transferability independently of the research question or method chosen. Data quality and completeness directly determine output reliability across methods: for statistical studies, incomplete transfer records introduce uncertainty into observed flow estimates; for modelling and simulation studies, input parameter quality directly determines the reliability of outputs. Studies relying on locally collected or system-specific data have limited generalisability than nationally administrative datasets. Full re-parameterisation is required when applying any model to a new data context.

### Implications for practice and policy

Efficient, safe and effective health systems depend on individual healthcare facilities with optimal capacity, location, bed occupancy and intended types of patients that correspond to their staff-mix and intended purpose. Therefore, analysing, understanding and influencing patient flows, demands and transfers are crucial for the successful development of health services. Patient flow across care settings requires extensive collaboration between service providers, presenting a significant challenge for healthcare professionals to oversee and guide these flows. To ensure efficient patient flow, health system professionals such as patient flow coordinators and service allocation officers can learn much from the use of modelling and simulation tools to understand system dynamics. Additionally, many health systems are increasingly focusing on integration and collaboration as the overarching policy objectives. For example, the UK’s NHS Long Term Plan highlights a shift toward Integrated Care Systems, promoting collaboration between primary and secondary care,^57^ Norway’s Coordination Reform similarly emphasises enhanced cooperation between primary and secondary care,^58^ aiming to ensure that the right treatment is provided at the right place and time. These systems and healthcare facilities must efficiently manage patient transfers to and from various parts of the healthcare and social care systems, such as care homes.

For practitioners and health system managers, the findings from this review suggest that method selection should begin with research questions and then an appraisal of system conditions, in terms of data availability, institutional capacity for modelling and the decision context. Statistical analysis is appropriate where routinely collected data are available and the goal is to understand current flows. Mathematical modelling is most valuable when theoretical relationships need to be formalised as a precursor to simulation. Computer simulation offers decision-support value when prospective scenario testing is required and when modelling teams can work in sustained collaboration with operational decision-makers. Practitioners also need to use the model critically, for example, the simplification of the reality in the modelling and simulation may limit the extent to which the models can contribute.

### Limitations of this review

Quality appraisal using the bespoke domain-based checklist showed variation in methodological reporting across the included studies. This made simulation and mathematical modelling studies generally score higher on method specification and results reporting. Statistical analysis studies, on the other hand, do not usually present a clear conceptual and structural framework and sometimes lack justification on variable selection and scored lower.

The review is subject to several additional limitations. The search was restricted to English language studies published 2013 onwards which might have led to missing a small number of relevant publications. The scope of this work is also constrained by the challenges inherent in conducting systematic reviews of this kind of literature. The studies were published across a broad spectrum of journals spanning healthcare, engineering, operational research and other fields, making interpretation and synthesis more challenging. Establishing a single standard for the assessment of study quality is challenging. The search was also limited to health and general scientific databases (MEDLINE, Embase, Web of Science, Google Scholar); engineering and computer science databases such as IEEE Xplore and ACM Digital Library were not searched. Given the interdisciplinary nature of operational research, relevant studies published in engineering or systems science venues may not have been captured, potentially under-representing technically sophisticated modelling approaches. In addition, a limitation of our review is that it does not systematically capture methodological contributions from computer science fields such as machine learning and data mining techniques. Although these methods may not be consistent with conventional statistical or operational research paradigms, they are becoming more significant tools for empirical analysis and can help guide the construction and calibration of simulation models in patient flow analysis. This omission is likely due to both the multidisciplinary fragmentation of the relevant literature and the inherent limitations of our systematic search technique. Future systematic reviews in this field might benefit from explicitly including computer science methodologies to offer a more comprehensive knowledge of the full spectrum of modelling tools available for patient flow analysis.

### Future research

Health systems are dynamic and evolving open systems that create challenges for capturing complete data about patient flows. Future research could benefit from exploring the use of hybrid models. For instance, Brice *et al* combined ABS and SDM and modelled patient flow from both clinical and operational perspectives.^53^ While our review found that most studies have focused on acute conditions and infectious diseases, there is a need for future work to emphasise chronic and non-communicable diseases. These diseases demand increased collaboration across specialised care, primary care and social care sectors. Furthermore, due to their long-term and wider societal impact, chronic diseases place a substantial strain on healthcare systems and public expenditures.

## Conclusion

This review revealed that analysing the patient flow between care settings or organisations can be effectively accomplished using a variety of methods, including statistical analysis, mathematical modelling and computer simulation. Each method tends to tackle distinct research aims but also has strengths and limitations in addressing various limitations in patient flow-related health service research. These findings suggest the potential benefits of applying more than one method for patient flow analysis. More published examples are required to use such methods to model the flow, demand and service utilisation of people with chronic and non-communicable diseases, as these conditions often involve multiple transitions between care settings over time.

## Supplementary material

10.1136/bmjopen-2025-115172 online supplemental file 1

10.1136/bmjopen-2025-115172 online supplemental file 2

10.1136/bmjopen-2025-115172 online supplemental file 3

## Data Availability

No data are available.
