# Supplementary material for: Methods for analysing patient flow between care settings: a systematic review
Source: BMJ Open. 2026 Aug 28;16(8):e115172. doi: 10.1136/bmjopen-2025-115172 (PMC13536093; doi:10.1136/bmjopen-2025-115172)
Supplement: Supplementary file 1 [file bmjopen-16-8-s001.pdf]

Date:13-March-2024

PRISMA-P 2015 checklist

Administrative information

1.Title: Methods for Analysing Patient Flow Between Care Settings: A Systematic Review

2.Authors: Fan Yang, Marianne Sundlisæter Skinner, Sujan Rijal, Alan Lovell, Lisa Victoria Burrell, Maren Kristine Raknes Sogstad, Rob Anderson

3.Support: Faculty of Medicine and Health Sciences, Norwegian University of Science and Technology (NTNU) and Norwegian Research Council

4.Introduction

Providing timely and integrated health services are two essential elements to realize the benefits of quality health care [1]. Many high-income countries are exploring the development of more integrated and patient-centred health systems to satisfy unmet needs by creating new models of care, such as intermediate care [2]. Such service models may also involve introducing different care pathways for specific patient groups or diseases, such as adding geriatric care units [3] and building short-stay crisis care units for mental health patients [4]. However, timely health services require access to health services in different service settings, – a seamless patient flow from the first contact to hospital admission, discharge, and further follow-up. In order to provide timely healthcare, in the last two decades there has been a growing research interest in mapping and changing patient flows to improve service provision and process management.

Whether it is in hospital or other care settings, a seamless patient flow across service settings can help avoid delay, improve quality of care, use the capacity effectively and reduce costs [5, 6]. For example, in the emergency department of two trusts in UK, they applied the Flow Cost Quality programme and both found sound evidence on the reduction of mortality, improved quality of care and reduction in length of stay and queue in the emergency care pathway [5]. Similar results were also found in the US that improved patient flow and reduced the waiting time and time during which there was dangerous and critical hospital overcrowding [7, 8]. In Murphy et al.'s research, they found that improved patient flow can not only reduce the waiting time, but also avoid the potential loss caused by reduced length of stay, and patients who left without being seen in the ED [8]. Though most studies focused on patient flow in emergency departments, primary care sectors, such as clinics, primary health care centre and other community-based care unit also seen reduced waiting time and efficiency with improved patient flow under high volume of patients [9-11]. However, the methods on analysing patient flow in primary care are poorly researched.

Earlier reviews on patient flow methods are usually classified into two sets: assessing patient flow to identify the deficiency and redesign to improve the patient flow. In one method systematic review published in 2013, authors listed seven techniques researched for analysing patient flow, including routine data analysis, modelling and simulation, and feedback from staff etc. As for changing patient flow, they mainly described the use of Lean approach and Six sigma [12]. Most reviews mainly focused on a single

modelling technique, such as machine learning [13], computer simulation [14] and artificial intelligence [15] to simulate patient flow in the hospital or emergency department. Only one review focused on patient flow methods applied in community care, and they also found that few models considered patient flow within systems of differing community services with most studies focus-sing on single services[16]. As the specialist health services and primary and community care services work closer and more collaboratively, methods on analysing and improving patient flow between different care settings or pathways needs to be updated and further synthesized.

To better understand methods used in analysing patient flows between service settings, this review will mainly address two research questions:

1. What are the main methods used for patient flow analysis to address health service research questions?
2. What are their relative strengths and limitations of these methods for answering different research or evaluation questions?

This review is part of a PhD project aiming to analyse and mapping patient flow in and out of Norwegian municipal in-patient acute units. The PhD project will focus on one of the work packages in a larger research project funded by the Research Council of Norway: Variations in trajectories, utilisation, and costs in Norwegian municipal in-patient acute care units (project number: 324959) [17].

5.Objectives

This review will primarily cover methods for analysing patient flows between care settings from a system perspective. An attempt will be made to identify current commonly used methods specific to patient flows across care systems and pathways. Critical appraisal will be used to evaluate what key issues or questions can be addressed appropriately by different methods. Along with analysis methods, the review will also analyse visualisation techniques from the selected studies.

6.Methods

Concept identification

As patient flows, patient pathway, clinical pathway and patient trajectories sometimes are used interchangeably in previous studies, it is necessary to distinguish different terms under contexts. The MeSH term "critical pathway" includes 17 entry terms including "clinical pathway", "care pathway" and "care map"[10]. These three terms were used interchangeably sharing the concepts developed by Vanhaecht in 2009 and accepted by the European Pathway Association, which is: "…a complex intervention for the mutual decision-making and organisation of care processes for a well-defined group of patients over a well-defined time frame."[18]. The pathway emphasised on the "intervention" with detailed step in a course of treatment or care, and it's a way of translating guidelines and evidence into local multidisciplinary plan of care [19]. The definition of patient trajectory is complex, as showed in Pescosolido's literature review, and

it has multifaced explanations under different time and contexts [20]. The common acceptable definition is provided by Alexander on his study about *Nurse-patient trajectory Framework* : "The term trajectory in health care can be defined as the assembling, scheduling, monitoring, and coordinating of all steps necessary to complete the work of patient care. The term trajectory refers not only to the pathophysiological process of a patient's disease state, but also refers to the total organization of work done throughout all nurse and patient interactions and refers to the impact of patient care processes on those interactions and the organization" [21]. Although in Pescosolido's review the definition of patient trajectory is hard to conclude, it clearly emphasizes that the trajectory should be around specific illness/disease career across life course, no matter the focus is on assembled nursing care or treatment [20]. As for patient flow, the definition is stated in NEJM journal catalyst, as "Patient flow is the movement of patients through a healthcare facility. It involves the medical care, physical resources, and internal systems needed to get patients from the point of admission to the point of discharge while maintaining quality and patient/provider satisfaction." [22]. Even though the terms have different definitions normatively, the practical intuitions made them used interchangeably by previous researchers [23-25].

This review will focus on patient "movement" across various care facilities; hence, patient flow will be the primary search term of the search. Due to the interchangeability of terminologies, the research will include papers that reference patient movement or transitions between different care settings, regardless of the terminology used.

Inclusion criteria

	**Inclusion criteria**	**Exclusion criteria**
**1**	Studies published from 2013 onwards	
**2**	In English language	
**3**	Studies that include patient flow analysis between any two or more **care settings/organisations**, such as hospitals, primary care clinics, outpatient/specialist clinics, long-term care facilities, or home health care.<br>AND<br>Studies that analyse patient flow between care settings using **any method**, such as workflow analysis, process mapping, time and motion studies, simulation modelling, data analysis, etc.	Studies that only analyse patient flow within one care setting, rather than between different care settings.<br>OR<br>Studies that do not involve patient flow analysis.
**3a**	OR are:<br>Reviews or Comparative studies on different methods for analysing patient flow (including reviews that analyse patient flows between care settings and organisations)	Protocols

Date:13-March-2024

Information sources

The strategies are assessed and implemented by the information specialist AL.

Five electronic databases will be searched: PubMed/Medline, Embase, CINAHL, the Cochrane Library and Controlled Trials Register, and the Health Management Information Consortium from 2013.01-2023.02

Search strategy:

#	Searches
1	(patient adj3 flow).ab,ti,kw.
2	(patient adj3 trajectory).ab,ti,kw.
3	(patient adj3 throughput).ab,ti,kw.
4	(patient adj3 route).ab,ti,kw.
5	(patient adj3 access*).ti.
6	(patient adj3 schedul*).ab,ti,kw.
7	(patient adj2 (discharge* or transfer*)).ti.
8	(patient adj3 transition).ab,ti,kw.
9	(referral adj3 network*).ab,ti,kw.
10	or/1-9

11 *computer simulation/ or *patient-specific modeling/

12 ((computer or "discrete event") adj simulation).ab,ti,kw.

13 (markov adj (decision* or chain* or model*)).ab,ti,kw.

14 ((mathematical or computer or statistic*) adj2 (model* or program*)).ab,ti,kw.

15 (operation* adj (manage* or research*)).ab,ti,kw.

16 optimi#ation.ab,ti,kw.

17 (system* adj3 model*).ab,ti,kw.

18 (queueing or queuing).ab,ti,kw.

19 (visual* adj3 (data* or method*)).ab,ti,kw.

20 exp *Methods/

21 exp data interpretation, statistical/ or exp models, statistical/

22 or/11-21

23 10 and 22

24 limit 23 to yr="2013 -Current"

7.Study records

data management: The records will be imported in the Rayyan software to share among authors for further screening.

selection and data collection process: To identify relevant research, information specialist AL will carry out the search, and three reviewers FY, RA and SR will first perform a pre-screening for the first 100 articles to reach a consensus of the inclusion and exclusion criteria. Then FY and SR will independently screen the records and discuss the uncertain records, and further decide on the inclusion of records. The Rayyan QCRI web-application will be used for the management of the data.

8. Data items:

Title, year, first author, objective/aims of study, study type, care settings, methods of analysing patient flow, methods of visualising patient flow, data type, data structure,

measured indicators

9. Outcomes and prioritization: Different methods used in analysing patient flows between different healthcare settings and pathways.

10. Synthesis

A well-structured table will maintain the information extracted from the selected papers. A thematic analysis will be conducted by:

(a) Identifying the information about the selected papers' methodologies and associated health service research issues addressed in chosen papers

(b) Organising the methodologies into subheadings and descriptive categories

(c) Developing analytical themes for strength and weakness for the methodologies.

Reference

1. World Health Organization (2020) Quality health services. https://www.who.int/news-room/fact-sheets/detail/quality-health-services. Accessed 11 Septermber 2025
2. Melis RJ, Olde Rikkert MG, Parker SG, et al. (2004) What is intermediate care? Bmj 329(7462):360–1. https://doi.org/10.1136/bmj.329.7462.360
3. Ijadi Maghsoodi A, Pavlov V, Rouse P, et al. (2022) Efficacy of acute care pathways for older patients: a systematic review and meta-analysis. European Journal of Ageing 19(4):1571–1585. https://doi.org/10.1007/s10433-022-00743-w
4. Anderson K, Goldsmith LP, Lomani J, et al. (2022) Short-stay crisis units for mental health patients on crisis care pathways: systematic review and meta-analysis. BJPsych Open 8(4):e144. https://doi.org/10.1192/bjo.2022.534
5. The Health Foundation (2013) Improving patient flow. https://www.health.org.uk/publications/improving-patient-flow. Accessed 11 Septermber 2025
6. Commonwealth Fund (2013) In Focus: Improving Patient Flow—In and Out of Hospitals and Beyond. https://www.commonwealthfund.org/publications/newsletter-article/focus-improving-patient-flow-and-out-hospitals-and-beyond#:~:text=Beyond%20%7C%20Commonwealth%20Fund-,In%20Focus%3A%20Improving%20Patient%20Flow%E2%80%94In%20and%20Out%20of%20Hospitals,to%20insurance%20coverage%20and%20care. Accessed 11 September 2025
7. Fleischman RJ, Kaji AH, Diaz VM, et al. (2015) A simple intervention to improve hospital flow from emergency department to inpatient units. JAMA Intern Med 175(2):289–90. https://doi.org/10.1001/jamainternmed.2014.6689
8. Murphy SO, Barth BE, Carlton EF, et al. (2014) Does an ED flow coordinator improve patient throughput? J Emerg Nurs 40(6):605–12. https://doi.org/10.1016/j.jen.2014.03.007
9. Potisek NM, Malone RM, Shilliday BB, et al. (2007) Use of patient flow analysis to improve patient visit efficiency by decreasing wait time in a primary care-based disease management programs for anticoagulation and chronic pain: a quality improvement study. BMC Health Serv Res 7):8. https://doi.org/10.1186/1472-6963-7-8
10. Bard JF, Shu Z, Morrice DJ, et al. (2016) Improving patient flow at a family health clinic. Health Care Manag Sci 19(2):170–91. https://doi.org/10.1007/s10729-014-9294-y
11. Fialho AS, Oliveira MD, and Sa AB (2011) Using discrete event simulation to compare the performance of family health unit and primary health care centre organizational models in

Portugal. BMC Health Serv Res (11):274. https://doi.org/10.1186/1472-6963-11-274
12. Silva Dd (2013) Improving patient flow across organisations and pathways. The Health Foundation 19. https://www.health.org.uk/publications/improving-patient-flow-across-organisations-and-pathways Accessed 29 Aug 2024
13. El-Bouri R, Taylor T, Youssef A, et al. (2021) Machine learning in patient flow: a review. Prog Biomed Eng (Bristol) 3(2):022002. https://doi.org/10.1088/2516-1091/abddc5
14. Mohiuddin S, Busby J, Savovic J, et al. (2017) Patient flow within UK emergency departments: a systematic review of the use of computer simulation modelling methods. BMJ Open 7(5):e015007. https://doi.org/10.1136/bmjopen-2016-015007
15. Cecula P, Yu J, Dawoodbhoy FM, et al. (2021) Applications of artificial intelligence to improve patient flow on mental health inpatient units - Narrative literature review. Heliyon 7(4):e06626. https://doi.org/10.1016/j.heliyon.2021.e06626
16. Palmer R, Fulop NJ, and Utley M (2018) A systematic literature review of operational research methods for modelling patient flow and outcomes within community healthcare and other settings. Health Syst (Basingstoke) 7(1):29–50. https://doi.org/10.1057/s41306-017-0024-9
17. Research Council of Norway. *Variations in trajectories, utilisation, and costs in Norwegian municipal in-patient acute care units*. 2021, https://prosjektbanken.forskningsradet.no/en/project/FORISS/324959?Kilde=FORISS&distribution=Ar&chart=bar&calcType=funding&Sprak=no&sortBy=date&sortOrder=desc&resultCount=30&offset=0&TemaEmne.2=Forskning+for+fornyelse+av+offentlig+sektor. Accessed 11 Septermber 2025
18. Vanhaecht K, De Witte K, Panella M, et al. (2009) Do pathways lead to better organized care processes? Journal of Evaluation in Clinical Practice 15(5):782–788. https://doi.org/10.1111/j.1365-2753.2008.01068.x
19. Lawal AK, Rotter T, Kinsman L, et al. (2016) What is a clinical pathway? Refinement of an operational definition to identify clinical pathway studies for a Cochrane systematic review. BMC Medicine 14(1):35. https://doi.org/10.1186/s12916-016-0580-z
20. Pescosolido BA, *Patient Trajectories*, in *The Wiley Blackwell Encyclopedia of Health, Illness, Behavior, and Society*. 2013. p. 1770–1777. https://doi.org/10.1002/9781118410868.wbehibs282
21. Alexander GL (2007) The nurse-patient trajectory framework. Stud Health Technol Inform 129(Pt 2):910–4. https://pmc.ncbi.nlm.nih.gov/articles/PMC3001102/
22. NEJM Catalyst (2018) What Is Patient Flow? Catalyst Carryover 4(1. https://doi.org/doi:10.1056/CAT.18.0289
23. Neuzillet C, Emery C, Teissier C, et al. (2022) Patient healthcare trajectories of intrahepatic cholangiocarcinoma in France: A nationwide retrospective analysis. The Lancet Regional Health – Europe 15. https://doi.org/10.1016/j.lanepe.2022.100324
24. Pinaire J, Azé J, Bringay S, et al. (2017) Patient healthcare trajectory. An essential monitoring tool: a systematic review. Health Information Science and Systems 5(1):1. https://doi.org/10.1007/s13755-017-0020-2
25. Olsen CF, Bergland A, Debesay J, et al. (2021) Patient Flow or the Patient's Journey? Exploring Health Care Providers' Experiences and Understandings of Implementing a Care Pathway to Improve the Quality of Transitional Care for Older People. Qualitative Health Research 31(9):1710–1723. https://doi.org/10.1177/10497323211003861
