# Supplementary material for: Methods for analysing patient flow between care settings: a systematic review
Source: BMJ Open. 2026 Aug 28;16(8):e115172. doi: 10.1136/bmjopen-2025-115172 (PMC13536093; doi:10.1136/bmjopen-2025-115172)
Supplement: Supplementary file 2 [file bmjopen-2025-115172.reviewer_comments.pdf]

PEER REVIEW HISTORY

BMJ Open publishes all reviews undertaken for accepted manuscripts. Reviewers are asked to complete a checklist review form and are provided with free text boxes to elaborate on their assessment. These free text comments are reproduced below.

ARTICLE DETAILS

Title (Provisional)

Methods for Analysing Patient Flow Between Care Settings: A Systematic Review

Authors

Yang, Fan; Skinner, Marianne Sundlisæter; Rijal, Sujan; Lovell, Alan; Burrell, Lisa Victoria; Sogstad, Maren; Anderson, Rob

---

VERSION 1 - REVIEW

**Reviewer**	**1**
**Name**	**Lopes, Manuel**
**Affiliation**	**University of Évora São João de Deus Higher School of Nursing**
**Date**	**10-Feb-2026**
**COI**	**None**

Dear Authors,

Firstly, I would like to thank you for the opportunity to learn about your work; secondly, I would like to congratulate you on the timeliness and relevance of the topic.

Having said that, I will now present a more detailed analysis for your consideration, in the certainty of the importance of the work carried out.

1. General assessment

This manuscript addresses an important topic: analytical methods for studying patient flow across care settings. The increasing complexity of health systems and the emphasis on integrated care make this a relevant and timely area of research.

The manuscript is clearly written and structured, and the classification of analytical methods is helpful. However, in its current form, the paper remains primarily descriptive and would benefit from stronger conceptual framing, deeper methodological critique, and clearer implications for research and policy.

Overall, the study is potentially publishable but requires substantive revision to strengthen its scholarly contribution.

2. Major concerns

2.1 Limited conceptual and theoretical framing

The manuscript focuses almost entirely on methodological classification but provides limited engagement with broader theoretical frameworks relevant to patient flow and system performance.

In particular, the paper would benefit from engagement with:

- complex adaptive systems theory,
- integrated care and care coordination frameworks,
- health system performance and resilience literature.

Without this framing, the review risks appearing as a technical catalogue of methods rather than a contribution to health services research theory.

2.2 Insufficient analytical depth in the synthesis

The synthesis of findings is largely descriptive. While methods are categorised and summarised, the review stops short of generating deeper insights such as:

- Which methods are most appropriate under different system conditions?
- Which methods produce evidence useful for decision-making versus academic modelling?
- Which methods have demonstrated real-world implementation impact?

A systematic review should move beyond describing studies to critically interpreting their significance.

2.3 Weak discussion of methodological limitations in the included literature

Although limitations are mentioned, the critique remains somewhat superficial.

Key issues that could be explored more critically include:

- external validity of simulation models,
- reproducibility and transparency of modelling studies,
- sensitivity to assumptions and parameter uncertainty,
- the gap between modelling outputs and policy implementation.

These issues are central in operational research applied to healthcare but are only briefly addressed.

2.4 Quality appraisal approach lacks transparency

The authors developed a bespoke domain-based checklist because no validated tool was available.

However:

- the checklist is not sufficiently described in the main text,
- scoring procedures and thresholds are unclear,
- it is not evident how quality assessment influenced conclusions.

This weakens confidence in the synthesis.

2.5 Search strategy may have introduced bias

Several choices may limit the comprehensiveness of the review:

- exclusion of non-English literature,
- restriction to studies published after 2013,
- limited database coverage.

These decisions are acknowledged but not sufficiently justified.

Given the interdisciplinary nature of operational research, relevant studies may exist in engineering and systems science databases that were not searched.

2.6 Limited engagement with heterogeneity

The included studies vary widely in:

- clinical contexts,
- healthcare systems,
- modelling approaches,
- data sources.

However, there is little attempt to analyse how these contextual differences influence:

- method selection,
- validity of results,
- transferability of findings.

This limits the interpretability of conclusions.

2.7 Implications for practice and policy are underdeveloped

One of the stated motivations of the review is to support policymakers and practitioners.

However, the discussion does not provide concrete guidance such as:

- when to use simulation versus statistical analysis,

how decision-makers should interpret modelling results,

- what data infrastructure is required to support these approaches.

This reduces the practical value of the paper.

3. Moderate concerns

3.1 Interpretation of novelty may be overstated

The authors state that this is the first review to identify and compare methods used to analyse patient flow across care settings.

This claim should be qualified more cautiously unless supported by a comprehensive scoping of adjacent literature (e.g., operational research reviews, health systems modelling reviews).

3.2 Descriptive repetition in Results and Discussion

Some sections of the Discussion restate findings rather than interpreting them, which reduces analytical depth.

3.3 Limited critical comparison between modelling approaches

Although the strengths and limitations of methods are summarised, the paper could better compare:

- predictive validity,

- computational requirements,

- interpretability for stakeholders,

- feasibility in real-world settings.

Such comparisons would significantly increase the value of the review.

4. Minor comments

- A conceptual framework diagram linking: research questions → data requirements → suitable methods would improve clarity.

- Some terminology could be standardised (e.g., modelling vs simulation vs statistical modelling).

- Greater emphasis on reproducibility and open modelling practices would be beneficial.

5. Strengths of the manuscript

Despite the concerns above, the paper has several important strengths:

- Clear research question and objectives

- Transparent selection process

- Useful synthesis tables

- Accessible explanation of modelling approaches

- Relevant and timely topic

These strengths provide a solid foundation for improvement.

6. Recommendation

Major revision

The manuscript is promising but requires substantial strengthening in:

- conceptual framing,
- critical analysis,
- methodological transparency,
- implications for practice and policy.

7. Final overall judgment

This manuscript is technically competent and well organised, but its contribution is currently more descriptive than analytical. With deeper interpretation, stronger theoretical framing, and clearer guidance for researchers and policymakers, it could become a valuable reference in the field.

**Reviewer**	**2**
**Name**	**Clay-Williams, Robyn**
**Affiliation**	**Macquarie University, Australian Institute of Health Innovation**
**Date**	**17-Apr-2026**
**COI**	**None**

Thank you for the opportunity to review your paper titled 'Method of analysing patient flow between care settings: a systematic review". The study sought to identify and compare methods for modelling patient flow between healthcare settings, but stops short of exploring outcomes associated with application of the methods (i.e. which methods are more effective in improving flow?)

Major point

The database search was conducted in Mar 2023 – there have likely been many relevant publications in the intervening 3 years and the search should be updated.

Minor points

Strengths/Limitations dotpoints. The dotpoints provide a summary of what you did, but do not really identify the key strengths/limitations of your study.

I believe you have actually produced a scoping review, rather than a systematic review (you are scoping which methods are used to analyse flow and how they are applied, rather than looking at outcomes associated application of the method). Your method is still fine for addressing your stated aims, but the title should be modified accordingly.

Typos

The is some repetition in the last sentence of the introduction.

There is a linking error in the last para of the data extraction section.

VERSION 1 - AUTHOR RESPONSE

Reviewer 1 comments

1.Major concerns

Reviewer's comment	Author's response
1.1 Limited conceptual and theoretical framing<br>The manuscript focuses almost entirely on methodological classification but provides limited engagement with broader theoretical frameworks relevant to patient flow and system performance. In particular, the paper would benefit from engagement with:<br>- complex adaptive systems theory,<br>- integrated care and care coordination frameworks,<br>- health system performance and resilience literature.<br>Without this framing, the review risks appearing as a technical catalogue of methods rather than a contribution to health services research theory.	Thank you for this suggestion. We have added a conceptual subsection in the Introduction 'Conceptualising patient flow between care settings' which engages with complex adaptive systems theory and integrated care and care coordination frameworks. This framing contextualises the methodological findings of the review within broader health systems theory.
1.2 Insufficient analytical depth in the synthesis<br>The synthesis of findings is largely descriptive. While methods are categorised and summarised, the review stops short of generating deeper insights such as:<br>- Which methods are most appropriate under different system conditions?<br>- Which methods produce evidence useful for decision-making versus academic modelling?<br>- Which methods have demonstrated real-world implementation impact?<br>A systematic review should move beyond describing studies to critically interpreting their significance.	Thank you for this observation. We have substantially expanded the analytical depth of the review. Section 3.4 and Table 4 now provide a structured method selection guide addressing system conditions, research aim fit, data requirements, output types, interpretability, Computational requirements, and real-world feasibility. Sections 4.2–4.4 of the Discussion critically interpret the significance of the identified patterns, including context-dependence, heterogeneity, and implications for practitioners and decision-makers.

1.3 Weak discussion of methodological limitations in the included literature<br>Although limitations are mentioned, the critique remains somewhat superficial. Key issues that could be explored more critically include:<br>- external validity of simulation models,<br>- reproducibility and transparency of modelling studies,<br>- sensitivity to assumptions and parameter uncertainty,<br>- the gap between modelling outputs and policy implementation.<br>These issues are central in operational research applied to healthcare but are only briefly addressed.	Thank you for this critique. Section 4.3 has been substantially expanded to address these issues. We now discuss: external validity constraints across all three method types; reproducibility as a systematic concern, noting that few studies provide analytical code or full parameter sets publicly; parameter uncertainty and the limited use of formal sensitivity analysis; and the gap between modelling outputs and policy implementation, including the conditions under which simulation evidence has and has not informed real service decisions.
1.4 Quality appraisal approach lacks transparency<br>The authors developed a bespoke domain-based checklist because no validated tool was available.<br>However:<br>- the checklist is not sufficiently described in the main text,<br>- scoring procedures and thresholds are unclear,<br>- it is not evident how quality assessment influenced conclusions.<br>This weakens confidence in the synthesis.	Thank you for raising this. We have expanded the description of the quality appraisal process and scoring criteria in Section 2.3. Section 4.5 explicitly discusses how quality findings contextualised the synthesis, including the observation that mathematical modelling and computer simulation studies generally scored higher on method specification than statistical analysis studies.
**1.5 Search strategy may have introduced bias**<br>Several choices may limit the comprehensiveness of the review:<br>- exclusion of non-English literature,<br>- restriction to studies published after 2013,<br>- limited database coverage.<br>These decisions are acknowledged but not sufficiently justified.<br>Given the interdisciplinary nature of operational research, relevant studies may exist in engineering and systems science databases that were not searched.	Thank you for this observation. We acknowledge these as genuine limitations and have made them explicit in the revised Strengths and Limitations section. The restriction to English-language studies, the 2013 publication cut-off, and the March 2023 search date are now stated as limitations. We also acknowledge in Section 4.5 that specialist operational research and systems engineering databases were not searched, which may have excluded relevant methodological literature from those fields
**1.6 Limited engagement with heterogeneity**	Thank you for this important point. We have added a dedicated Section 4.2 —

The included studies vary widely in:<br>- clinical contexts,<br>- healthcare systems,<br>- modelling approaches,<br>- data sources.<br>However, there is little attempt to analyse how these contextual differences influence:<br>- method selection,<br>- validity of results,<br>- transferability of findings.<br>This limits the interpretability of conclusions.	'Heterogeneity and Context-Dependence'. This section analyses how differences in clinical context, healthcare system structure, and modelling approach shape method selection, validity of results, and transferability of findings.
1.7 Implications for practice and policy are underdeveloped<br>One of the stated motivations of the review is to support policymakers and practitioners.<br>However, the discussion does not provide concrete guidance such as:<br>- when to use simulation versus statistical analysis,<br>how decision-makers should interpret modelling results,<br>- what data infrastructure is required to support these approaches.<br>This reduces the practical value of the paper.	Thank you for this suggestion. Section 4.4 and Table 4 have been substantially expanded to provide concrete guidance for practitioners and health system managers. This includes decision criteria for method selection based on research aim, data availability, and system complexity; guidance on critically interpreting modelling outputs, including awareness of model simplifications and their practical limits; and discussion of the data infrastructure required to support each method family.

2. Moderate concerns

Reviewer's comment	Author's response
2.1 Interpretation of novelty may be overstated<br>The authors state that this is the first review to identify and compare methods used to analyse patient flow across care settings.<br>This claim should be qualified more cautiously unless supported by a comprehensive scoping of adjacent literature (e.g., operational research reviews, health systems modelling reviews).	Thank you for your comment. We agree that this statement is difficult to substantiate without a comprehensive scoping of adjacent literature across operational research, health systems modelling, and related fields. We have therefore removed it and instead begin the Discussion with a direct summary of findings.

2.2 Descriptive repetition in Results and Discussion<br>Some sections of the Discussion restate findings rather than interpreting them, which reduces analytical depth.	Thank you for the comment. We reviewed the Results and Discussion sections and identified instances where findings were restated rather than interpreted. We have revised the section accordingly; please see Section 4.1.
2.3 Limited critical comparison between modelling approaches<br>Although the strengths and limitations of methods are summarised, the paper could better compare:<br>- predictive validity,<br>- computational requirements,<br>- interpretability for stakeholders,<br>- feasibility in real-world settings.<br>Such comparisons would significantly increase the value of the review.	Thank you for the suggestion. We agree that a structured comparison of methods across these dimensions would strengthen the review's practical contribution. Accordingly, we have added Table 4 to provide a comparative overview of the methods.

3. Minor comments

Reviewer's comment	Author's response
- A conceptual framework diagram linking: research questions → data requirements → suitable methods would improve clarity.	Thank you for this suggestion. We have added Table 4, which provides a method selection guide linking system conditions and research aims to data requirements and suitable methods. Together with the Sankey diagram (Fig. 4), which maps method–topic relationships across included studies, we believe these additions address the spirit of this suggestion without introducing a redundant diagram.
- Some terminology could be standardised (e.g., modelling vs simulation vs statistical modelling).	Thank you for the comment. We have standardized the terms.<br>Section 3.1.3 heading -> changed "Simulation modelling" to "Computer Simulation"<br>Table 3 header ->change "Simulation" to "Computer simulation"<br>Section 3.1 -> change "computer simulation modelling" to "computer simulation"
- Greater emphasis on reproducibility and open modelling practices would be beneficial.	Thank you for the comment. We had added a paragraph in section 4.3 inferring the reproducibility and modelling practices.

Reviewer 2 comment

1. Major point

Reviewer's comment	Author's response
The database search was conducted in Mar 2023 – there have likely been many relevant publications in the intervening 3 years and the search should be updated.	Thank you for the comment. We've updated the search to 10 June 2026.

2. Minor points

Reviewer's comment	Author's response
Strengths/Limitations dotpoints. The dotpoints provide a summary of what you did, but do not really identify the key strengths/limitations of your study.	Thank you for this observation. We have further deepened the appraisal and comparison of methods and expanded the discussion accordingly. A key strength of the current review is that it provides a detailed method selection guide for practitioners and health system managers. We have also added a dedicated section in the Discussion addressing the limitations of the included studies.
I believe you have actually produced a scoping review, rather than a systematic review (you are scoping which methods are used to analyse flow and how they are applied, rather than looking at outcomes associated application of the method). Your method is still fine for addressing your stated aims, but the title should be modified accordingly.	Thank you for this important methodological observation. We acknowledge that our primary aim — to identify and map methods used for patient flow analysis — shares characteristics with scoping review methodology. However, our review extends beyond a scoping approach through having a comprehensive comparison between methods.

3. Typos

Reviewer's comment	Author's response
The is some repetition in the last sentence of the introduction.<br>There is a linking error in the last para of the data extraction section.	Thank you for these observations. We have corrected the repetition and linking error in the last sentence of the Introduction and last paragraph of the data extraction section, respectively.
