# Supplementary material for: Methods for analysing patient flow between care settings: a systematic review
Source: BMJ Open. 2026 Aug 28;16(8):e115172. doi: 10.1136/bmjopen-2025-115172 (PMC13536093; doi:10.1136/bmjopen-2025-115172)
Supplement: Supplementary file 3 [file bmjopen-16-8-s002.docx]

**Medline**

Database(s): **Ovid MEDLINE(R) ALL** 1946 to March 13, 2023
Search Strategy:

Search date 14 March

| **#** | **Searches** | **Results** |
| --- | --- | --- |
| 1 | (patient* adj2 flow*).ab,ti,kw. | 8705 |
| 2 | (patient* adj2 trajector*).ab,ti,kw. | 1517 |
| 3 | (patient* adj2 throughput*).ab,ti,kw. | 827 |
| 4 | (patient* adj2 route*).ab,ti,kw. | 955 |
| 5 | (patient* adj2 access*).ti,ab. /freq=3 | 392 |
| 6 | (patient* adj2 schedul*).ti,ab. /freq=3 | 192 |
| 7 | (patient* adj2 (discharge* or transfer*)).ti,ab. /freq=3 | 2429 |
| 8 | ((patient* or treatment* or care*) adj2 transition*).ti,ab. /freq=3 | 1845 |
| 9 | ((patient* or treatment* or care*) adj2 pathway*).ti,ab. /freq=3 | 1645 |
| 10 | (referral* adj2 network*).ab,ti,kw. | 523 |
| 11 | or/1-10 | 18800 |
| 12 | *computer simulation/ or *patient-specific modeling/ | 40505 |
| 13 | ((computer or "discrete event") adj simulation*).ab,ti,kw. | 26659 |
| 14 | (markov adj (decision* or chain* or model*)).ab,ti,kw. | 20800 |
| 15 | ((mathematical or computer or statistic*) adj2 (model* or program*)).ab,ti,kw. | 121151 |
| 16 | (operation* adj (manage* or research*)).ab,ti,kw. | 2698 |
| 17 | optimi#ation.ab,ti,kw. | 192047 |
| 18 | (system* adj3 model*).ab,ti,kw. | 120270 |
| 19 | (queueing or queuing).ab,ti,kw. | 918 |
| 20 | (visual* adj3 (data* or method*)).ab,ti,kw. | 27718 |
| 21 | exp *Methods/ or method*.ti,ab. /freq=4 | 427318 |
| 22 | exp *data interpretation, statistical/ or exp *models, statistical/ | 64403 |
| 23 | or/12-22 | 969660 |
| 24 | 11 and 23 | 1091 |
| 25 | limit 24 to yr="2013 -Current" | 723 |

**Embase**

Database(s): **Embase** 1974 to 2023 March 13
Search Strategy:

Search date 14 March

| **#** | **Searches** | **Results** |
| --- | --- | --- |
| 1 | (patient* adj2 flow*).ab,ti,kw. | 14116 |
| 2 | (patient* adj2 trajector*).ab,ti,kw. | 2593 |
| 3 | (patient* adj2 throughput*).ab,ti,kw. | 1412 |
| 4 | (patient* adj2 route*).ab,ti,kw. | 1624 |
| 5 | (patient* adj3 access*).ti,ab. /freq=3 | 1537 |
| 6 | (patient* adj2 (discharge* or transfer*)).ti,ab. /freq=3 | 5072 |
| 7 | ((patient* or treatment* or care*) adj2 transition*).ti,ab. /freq=3 | 3266 |
| 8 | ((patient* or treatment* or care*) adj2 pathway*).ti,ab. /freq=3 | 2947 |
| 9 | (referral adj3 (network* or system* or pathway*)).ab,ti,kw. | 8299 |
| 10 | or/1-9 | 40195 |
| 11 | *computer simulation/ or *patient-specific modeling/ | 82731 |
| 12 | ((computer or "discrete event") adj simulation).ab,ti,kw. | 14541 |
| 13 | (markov adj (decision* or chain* or model*)).ab,ti,kw. | 28360 |
| 14 | ((mathematical or computer or statistic*) adj2 (model* or program*)).ab,ti,kw. | 149846 |
| 15 | (operation* adj (manage* or research*)).ab,ti,kw. | 3227 |
| 16 | optimi#ation.ab,ti,kw. | 245566 |
| 17 | (system* adj3 model*).ab,ti,kw. | 138740 |
| 18 | (queueing or queuing).ab,ti,kw. | 1120 |
| 19 | (visual* adj3 (data* or method*)).ab,ti,kw. | 38967 |
| 20 | *statistical analysis/ | 20092 |
| 21 | *mathematical model/ | 26190 |
| 22 | method*.ti,ab. /freq=4 | 471419 |
| 23 | or/11-22 | 1135888 |
| 24 | 10 and 23 | 1843 |
| 25 | limit 24 to yr="2013 -Current" | 1332 |
| 26 | limit 25 to conference abstract status | 581 |
| 27 | 25 not 26 | 751 |

**Web of science**

Search date 15 March

https://www.webofscience.com/wos/woscc/summary/3a923ec4-444e-42cb-9921-02f3cf2464f3-790578b6/recently-added/1

1. AB=("patient flow" OR "flow of patients")
2. TS=(method)
3. TI=(method*)
4. #2 OR #3
5. #1 AND #4
6. #5 and **2023** or **2022** or **2021** or **2020** or **2019** or **2018** or **2017** or **2016** or **2015** or **2014** or **2013** (Publication Years) and **Article** or **Review Article** (Document Types) and **Emergency Medicine** or **Medicine General Internal** or **Surgery** (Exclude – Web of Science Categories)

Total hits = 690

**Google Scholar**

Search date 15 March

("patient flow" or "flow of patients") and (method or model or simulation). 2013-2023

Took the first 50 from “About 1,970 results”

**UPDATE SEARCH**

12 June 2026

Database(s): **Ovid MEDLINE(R) ALL** 1946 to June 11, 2026
Search Strategy:

| **#** | **Searches** | **Results** |
| --- | --- | --- |
| 1 | (patient* adj2 flow*).ab,ti,kw. | 10269 |
| 2 | (patient* adj2 trajector*).ab,ti,kw. | 2698 |
| 3 | (patient* adj2 throughput*).ab,ti,kw. | 1022 |
| 4 | (patient* adj2 route*).ab,ti,kw. | 1129 |
| 5 | (patient* adj2 access*).ti,ab. /freq=3 | 525 |
| 6 | (patient* adj2 schedul*).ti,ab. /freq=3 | 245 |
| 7 | (patient* adj2 (discharge* or transfer*)).ti,ab. /freq=3 | 3045 |
| 8 | ((patient* or treatment* or care*) adj2 transition*).ti,ab. /freq=3 | 2688 |
| 9 | ((patient* or treatment* or care*) adj2 pathway*).ti,ab. /freq=3 | 2463 |
| 10 | (referral* adj2 network*).ab,ti,kw. | 752 |
| 11 | or/1-10 | 24536 |
| 12 | *computer simulation/ or *patient-specific modeling/ | 43222 |
| 13 | ((computer or "discrete event") adj simulation*).ab,ti,kw. | 29049 |
| 14 | (markov adj (decision* or chain* or model*)).ab,ti,kw. | 26455 |
| 15 | ((mathematical or computer or statistic*) adj2 (model* or program*)).ab,ti,kw. | 143213 |
| 16 | (operation* adj (manage* or research*)).ab,ti,kw. | 3247 |
| 17 | optimi#ation.ab,ti,kw. | 293949 |
| 18 | (system* adj3 model*).ab,ti,kw. | 144996 |
| 19 | (queueing or queuing).ab,ti,kw. | 1167 |
| 20 | (visual* adj3 (data* or method*)).ab,ti,kw. | 37277 |
| 21 | exp *Methods/ or method*.ti,ab. /freq=4 | 528046 |
| 22 | exp *data interpretation, statistical/ or exp *models, statistical/ | 72145 |
| 23 | or/12-22 | 1230958 |
| 24 | 11 and 23 | 1477 |
| 25 | limit 24 to yr="2023 -Current" | 413 |

Database(s): **Embase** 1974 to 2026 June 10
Search Strategy:

| **#** | **Searches** | **Results** |
| --- | --- | --- |
| 1 | (patient* adj2 flow*).ab,ti,kw. | 17615 |
| 2 | (patient* adj2 trajector*).ab,ti,kw. | 4439 |
| 3 | (patient* adj2 throughput*).ab,ti,kw. | 1854 |
| 4 | (patient* adj2 route*).ab,ti,kw. | 2080 |
| 5 | (patient* adj3 access*).ti,ab. /freq=3 | 2105 |
| 6 | (patient* adj2 (discharge* or transfer*)).ti,ab. /freq=3 | 6596 |
| 7 | ((patient* or treatment* or care*) adj2 transition*).ti,ab. /freq=3 | 4643 |
| 8 | ((patient* or treatment* or care*) adj2 pathway*).ti,ab. /freq=3 | 4593 |
| 9 | (referral adj3 (network* or system* or pathway*)).ab,ti,kw. | 12980 |
| 10 | or/1-9 | 55945 |
| 11 | *computer simulation/ or *patient-specific modeling/ | 88318 |
| 12 | ((computer or "discrete event") adj simulation).ab,ti,kw. | 15810 |
| 13 | (markov adj (decision* or chain* or model*)).ab,ti,kw. | 35338 |
| 14 | ((mathematical or computer or statistic*) adj2 (model* or program*)).ab,ti,kw. | 178993 |
| 15 | (operation* adj (manage* or research*)).ab,ti,kw. | 3956 |
| 16 | optimi#ation.ab,ti,kw. | 362726 |
| 17 | (system* adj3 model*).ab,ti,kw. | 165627 |
| 18 | (queueing or queuing).ab,ti,kw. | 1395 |
| 19 | (visual* adj3 (data* or method*)).ab,ti,kw. | 52298 |
| 20 | *statistical analysis/ | 22617 |
| 21 | *mathematical model/ | 30186 |
| 22 | method*.ti,ab. /freq=4 | 593149 |
| 23 | or/11-22 | 1444479 |
| 24 | 10 and 23 | 2885 |
| 25 | limit 24 to yr="2023 -Current" | 965 |
| 26 | limit 25 to conference abstract status | 384 |
| 27 | 25 not 26 | 581 |

**Web of Science**

1. AB=("patient flow" OR "flow of patients")
2. TS=(method)
3. TI=(method*)
4. #2 OR #3
5. #1 AND #4

#5 and **2026** or **2025** or **2024** or **2023** (Publication Years) and **Article** or **Review Article** (Document Types) and **Emergency Medicine** or **Medicine General Internal** or **Surgery** (Exclude – Web of Science Categories)

Total hits = 292

**Google Scholar**

("patient flow" or "flow of patients") and (method or model or simulation). 2023-2026

Took the first 50 from “About785 results”

**Total found = 1336**

**After deduping = 956**
