# Supplementary material for: Methods for analysing patient flow between care settings: a systematic review
Source: BMJ Open. 2026 Aug 28;16(8):e115172. doi: 10.1136/bmjopen-2025-115172 (PMC13536093; doi:10.1136/bmjopen-2025-115172)
Supplement: Supplementary file 4 [file bmjopen-16-8-s003.docx]

**Supplementary information S3** Characteristics of studies included from full text screening

| **No.** | **Country** | **Aim** | **Patient flow-related health service topic** | **Setting/Organisations** | **Method** | **Method output** |
| --- | --- | --- | --- | --- | --- | --- |
| 1 | United States^27^ | To understand the prevalence of multidrug-resistant organism (MDRO) infections among nursing home (NH) residents and the potential for their spread between NHs and acute care hospitals (ACHs). | Spread of infection between care settings | 1. Nursing homes 2. Acute care hospitals | Descriptive and group comparison test | Infection prevalence |
| 2 | Italy^28^ | To introduce Bayesian exponential random graph modelling, discuss model specification and parameter estimation in the context of interhospital referral network as the empirical setting. | Referral network development | 1. Hospital 2. Hospital | 1. Descriptive and group comparison test 2. Statistical modelling | Referral propensity |
| 3 | Ethiopia^29^ | To explore the documentation of the referral management process and the mechanism, and quality of referrals between the health centre and hospital | Patient transfer volume and outcome | 1. Health centre  2. Hospital | Descriptive and group comparison test | Incidence of unnecessary transfers |
| 4 | United Kingdom^30^ | To illustrate the false economy of seeking to eliminate delayed transfers of care (DTOCs) and to show that a certain amount of ‘bed blockage’ is beneficial to reducing the total costs across the continuum of care. | Capacity planning; Economic budget control | 1. Acute care 2. Community care | Mathematical modelling | Cost-optimal delayed patient transfer amount |
| 5 | United Kingdom^31^ | To estimate the theoretical cost-optimal capacity requirement for ‘step down’ intermediate care services within a major healthcare system in England | Capacity planning Economic budget control | 1. Acute care 2. Intermediate care | 1. Mathematical modelling 2. Discrete event simulation | Demand for care Cost-optimal delayed patient transfer amount |
| 6 | Lithuania^32^ | To identify the importance of the partnership between the public and private healthcare institutions to improve interhospital patient transfers. | Patient transfer volume and outcome | 1. Ambulance 2. First hospital 3. Tertiary hospital | Descriptive and group comparison test | Patient transfer numbers and percentages |
| 7 | United States^33^ | To present a method for accurately imputing unobserved transfers, thereby providing an accurate patient transfer network based on partially observed data. | Referral network development | 1. Hospital 2. Hospital | Statistical modelling | Patient transfer network |
| 8 | China^34^ | To evaluate the empirical effects of vertical integration reform on primary healthcare institutions (PHIs) in China and examine variations across integration intensity (tight integration vs. loose collaboration) | Patient transfer volume and outcome | 1.Primary healthcare institutions (township health centre, community health centre) 2. Hospital | 1. Descriptive and group comparison test 2. Regression analysis | Patient transfer numbers and percentage |
| 9 | United States^35^ | Using the Regional Healthcare Ecosystem Analyst (RHEA) to simulate patient flow among facilities in the region and better understand the spread and control of infectious diseases. | Spread of infection between care settings Referral network development Capacity planning | 1. Hospital 2. Other facility | Agent-based simulation | Infection transmission among facilities Patient transfer network Length of stay Probability of readmission Bed capacity |
| 10 | China^36^ | To propose a methodology that can automatically optimise the policy control variables to improve the multi-objective system performances in the hierarchical healthcare delivery system. | Capacity planning | 1. Community Health Centre 2. General Hospital | 1. Mathematical modelling 2. Discrete event simulation | Throughput Length of stay Leave without being seen (LWBS) rate |
| 11 | United Kingdom^37^ | To analyse patient flow through a new care pathway and compare with baseline for Hepatitis C patients. | Spread of infection between care settings | 1. GP 2. Specialist care | Descriptive and group comparison test | Patient transfer numbers and percentages |
| 12 | United States^38^ | To develop a simulation model of patient flow to understand the relationship between capacity and demand, and to investigate the impact on performance measures such as average wait times for long-term care patients. | Capacity planning | 1. Acute care hospital 2. Long-term care facilities (including nursing homes and intermediate care facilities) | Discrete event simulation | Demand of care  Bed capacity Waiting time Resource utilisation |
| 13 | Germany^39^ | To derive a strategy to counteract the spread of multi-drug-resistant Enterobacteriaceae (MDR-E) at the regional healthcare network level. | Spread of infection between care settings Referral network development | 1. Hospitals 2. Hospitals 3. Community | Mathematical modelling (Network models for epidemiology modelling) | Infection prevalence Patient transfer network |
| 14 | Canada^40^ | To describe the characteristics and associated outcomes among interhospital transferred and non-transferred Canadian patients with acute cardiovascular disease | Patient transfer volume and outcome | 1. Hospital 2. Hospital | 1. Descriptive and group comparison test 2. Regression analysis | Mortality rate |
| 15 | China^41^ | To evaluate the patient flow distribution using the proposed simulation-based optimisation method. | Referral network development Capacity planning | 1. Community health care centres (CHC)  2. General hospital (GH) | 1. Mathematical modelling  2. Discrete event simulation | Throughput System profit Patient transfer network |
| 16 | United Kingdom^42^ | To present a hybrid simulation modelling framework containing both agent-based simulation and system dynamics to capture both depression progression and patient flow. | Capacity planning | 1. GP 2. Inpatient care | 1.Agent-based simulation 2.System Dynamics | Depression prevalence rate  Demand of care  Service capacities |
| 17 | United States^43^ | To evaluate the transfer of injured patients in a mature, inclusive trauma system. | Patient transfer volume and outcome | 1. Level 1 trauma centre 2. Level 3 trauma centre | 1. Descriptive and group comparison test 2. Regression analysis | Length of stay  Mortality rate |
| 18 | The Netherlands^44^ | To describe the flow of patients with Traumatic spinal cord injury (TSCI) through the prehospital, hospital, and rehabilitation settings and to relate treatment outcomes to emergency medical services (EMS) transport locations and surgery timing. | Patient transfer volume and outcome | 1. EMS 2. Hospital 3. Community rehabilitation | Descriptive and group comparison test | Mortality rate |
| 19 | United Kingdom^45^ | To identify current capacity bottlenecks that affect patient flow, future capacity requirements in the presence of increased admissions, the impact of co-location and pooling of the acute and rehabilitation units and the impact of patient subgroups on capacity requirements. | Capacity planning | 1. Acute ward  2. Community rehabilitation | Discrete event simulation | Length of stay  Bed capacity |
| 20 | United States^46^ | To determine the incidence of clinically necessary transfer within a regional transfer network. To identify factors associated with unnecessary transfers that could be targeted for interventions at the level of facilities and delivery systems | Patient transfer volume and outcome | 1. Acute care referring hospitals 2. Tertiary referral centre | 1.Descriptive and group comparison test 2. Regression analysis | Incidence of unnecessary transfers |
| 21 | UK^47^ | To describe the flow of patients across multiple mental health services in the system and explore different scenarios relating to future demand. | Capacity planning | 1. Primary care  2. Outpatient care  3. Inpatient care | Discrete event simulation (Discrete time simulation) | Wait list size, occupancy, leave without being seen(LWBS) rate; |
| 22 | China^48^ | To investigate how differential health insurance reimbursement rates can be leveraged to guide patient flow. | Patient transfer volume and outcome | 1. Primary hospital  2. Secondary and tertiary hospitals | Agent-based simulation | Hospital utilisation rate |
| 23 | UK^49^ | To map NHS Talking Therapies care pathways. | Patient transfer volume and outcome | 1. Hospital  2. Hospital | Descriptive and group comparison test | Wait time |
| 24 | Canada^50^ | To investigate interhospital patient transfer policies to alleviate hospital congestion and inequity in the distribution of patients across the health system. | Capacity planning | 1. Hospital  2. Hospital | Mathematical modelling | Length of stay |
